# Synthetic Glycans Reveal Determinants of Antibody Functional Efficacy against a Fungal Pathogen

**DOI:** 10.1021/acsinfecdis.3c00447

**Published:** 2023-10-19

**Authors:** Conor J. Crawford, Lorenzo Guazzelli, Scott A. McConnell, Orla McCabe, Clotilde d’Errico, Seth D. Greengo, Maggie P. Wear, Anne E. Jedlicka, Arturo Casadevall, Stefan Oscarson

**Affiliations:** † Centre for Synthesis and Chemical Biology, University College Dublin, Belfield D04 V1W8, Dublin 4, Ireland; ‡ Department of Molecular Microbiology and Immunology, Johns Hopkins Bloomberg School of Public Health, 615 North Wolfe Street, Baltimore, Maryland 21205, United States

**Keywords:** glycans, antibodies, epitopes, immunology, vaccines, *Cryptococcus neoformans*

## Abstract

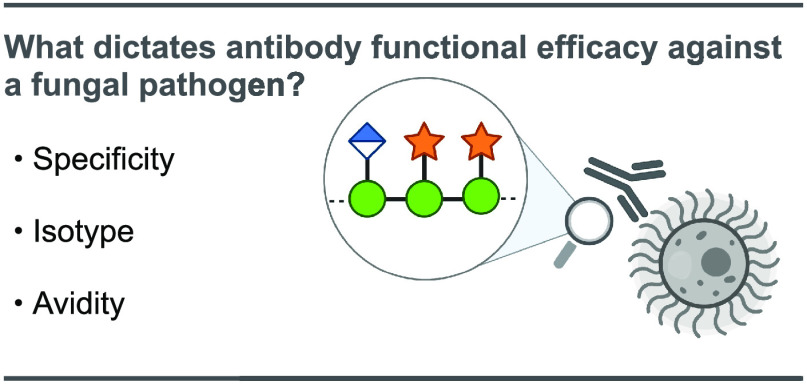

Antibodies play a vital role in the immune response to infectious diseases and can be administered passively to protect patients. In the case of *Cryptococcus neoformans*, a WHO critical priority fungal pathogen, infection results in antibodies targeting capsular glucuronoxylomannan (GXM). These antibodies yield protective, non-protective, and disease-enhancing outcomes when administered passively. However, it was unknown how these distinct antibodies recognized their antigens at the molecular level, leading to the hypothesis that they may target different GXM epitopes. To test this hypothesis, we constructed a microarray containing 26 glycans representative of those found in highly virulent cryptococcal strains and utilized it to study 16 well-characterized monoclonal antibodies. Notably, we found that protective and non-protective antibodies shared conserved reactivity to the M2 motif of GXM, irrespective of the strain used in infection or GXM-isolated to produce a conjugate vaccine. Here, only two antibodies, 12A1 and 18B7, exhibited diverse trivalent GXM motif reactivity. IgG antibodies associated with protective responses showed cross-reactivity to at least two GXM motifs. This molecular understanding of antibody binding epitopes was used to map the antigenic diversity of two *Cryptococcus neoformans* strains, which revealed the exceptional complexity of fungal capsular polysaccharides. A multi-GXM motif vaccine holds the potential to effectively address this antigenic diversity. Collectively, these findings underscore the context-dependent nature of antibody function and challenge the classification of anti-GXM epitopes as either “protective” or “non-protective”.

Advancing molecular immunology necessitates defining the binding and functional properties of antibodies, including understanding how different characteristics, such as epitope specificity and isotype function, contribute to protective efficacy.^1−3^ This knowledge forms the basis for the rational design of vaccines and monoclonal antibody-based therapies. While vaccines against bacteria and viruses have been successfully developed, there are currently no commercial vaccines against fungi. Given the rising global temperatures and increasing immunocompromised populations, fungal infections may become more prevalent.^4,5^ Recognizing the severity of this issue, the World Health Organization in 2022 established its first pathogen priority list for fungi,^6^ placing *Cryptococcus neoformans* in the top “critical priority group”.^6^ Serological evidence suggests *C. neoformans* infections are common, with immunocompetent hosts often clearing the infection while immunocompromised individuals face over 600,000 deaths annually.^7−9^ Currently, there is limited molecular understanding of host–microbe interactions between *C. neoformans* and the mammalian immune system, which is crucial information for developing effective therapies.^10,11^ The protective potential of antibodies against cryptococcal infections is linked to their unique capsular binding patterns, which, in turn, are functions of their epitope specificity. However, there is no precise knowledge of how these antibodies bind their epitopes, leaving the molecular basis for their distinct patterns of binding to cryptococcal cells unexplored.

The *C. neoformans* polysaccharide capsule is crucial for virulence.^12,13^ However, the cryptococcal capsule is a complex structure composed of a variety of glycans and glycoproteins.^14−17^ The main component of the capsule is glucuronoxylomannan (GXM), a complex and heterogeneous polysaccharide with distinct structural motifs (Figure 1).^18^ This lack of a defined repeating unit sets the *C. neoformans* capsule apart from bacterial capsules and aligns it more closely with polysaccharides found in algae and plants.^19,20^ Unlike bacterial serotypes, GXM motifs occur simultaneously in ratios depending that depend on the strain and environmental conditions.^21,22^ This complexity results in several open questions regarding the biosynthesis, assembly and structure of the cryptococcal capsule.^23−30^ The structure of GXM consists of an α-1,3-mannan backbone decorated with branches of β-1,2- and β-1,4-xylose and β-1,2-glucuronic acid (Figure 1). The motif is determined by the xylose branching pattern, which has been historically associated with serotype classification. However, the current conflation of these two aspects, serotype and glycan structure, is characterized by significant uncertainty.^15,31−33^ Additionally, the molecular diversity of GXM is further increased by the presence of a 6-*O*-acetylation pattern on the mannan backbone, which often plays a significant role in antibody binding and virulence.^34,35^

**Figure 1:**
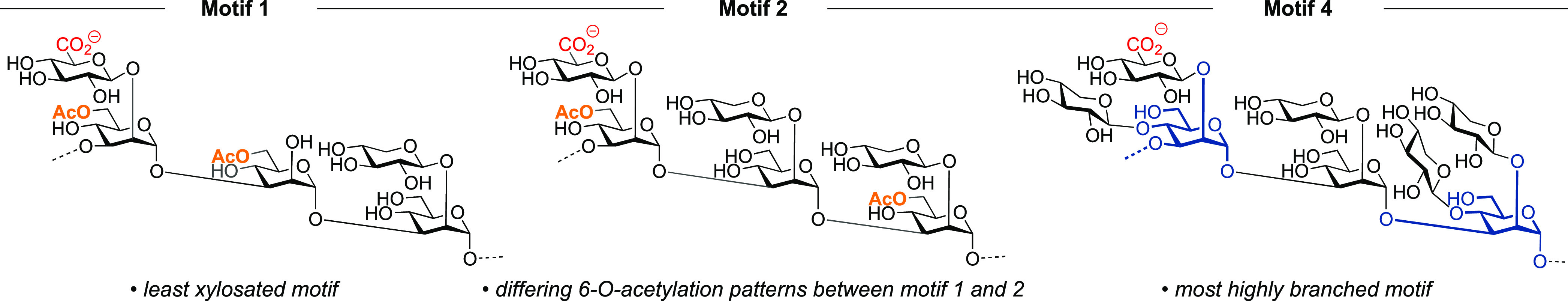
Common structural motifs found in glucuronoxylomannan (GXM).

Given the success of bacterial conjugate vaccines, the capsular polysaccharide of *C. neoformans* is a prime target for developing conjugate vaccines and monoclonal antibodies. Currently, a library of monoclonal antibodies exists which produce complex outcomes when used in passive immunization, including protection, non-protection and disease enhancement.^36,37^ This has led to the hypothesis that different GXM epitopes can elicit protective or non-protective antibody responses, a phenomenon well established for protein-based antigens.^38,39^ However, the plausibility of this hypothesis in the context of GXM-specific antibodies is not clear. Understanding the link between the epitope(s) recognized by an antibody and the functional efficacy of the antibody in the context of cryptococcal immunity requires specific knowledge of the glycotopes. Until now, specificity profiling of these mAbs has been limited to heterogeneous mixtures of GXM glycans from biological sources. Chemical synthesis, on the other hand, is distinct for its ability to produce well-defined glycans,^40^ which can be used to precisely map epitopes of antibodies (SI Scheme 2).^34,39,41−47^ Therefore, we synthesized a comprehensive library of 26 structurally diverse GXM oligosaccharides, representing the major motifs found in pathogenic cryptococcal strains.^48−50^ These synthetic glycans contained differences in xylose branching, *O*-acetylation, and length, enabling precise molecular investigation into the determinants of glycan–antibody interactions. Printing these glycans on a microarray allowed for a high-throughput means to analyze the binding specificities of 16 monoclonal antibodies (mAbs). Overall, we discovered that antibody function against this fungal pathogen cannot be attributed to any single factor in isolation, but instead is intricately linked to a combination of antibody characteristics. As a result, we propose a context-dependent framework that considers the antigenic diversity of this fungal pathogen, which is known to vary considerably across strains. This antigenic diversity raises a considerable challenge for both vaccine and therapeutic mAb design. We propose that it may be overcome by using multi-GXM motif conjugate vaccines formulations and antibody cocktails, respectively.

## Results and Discussion

### Chemical Synthesis and Printing of a GXM Glycan Microarray

A convergent building block approach was used to access protected GXM oligosaccharides, primarily utilizing disaccharide building blocks with benzyl ethers as permanent protecting groups and chloroacetyl esters as temporary protecting groups.^43,44,51−53^ This strategy enabled access to the desired acetylated target structures, with the acetylation patterns for M1 and M2 motif GXM chosen based on earlier investigations (Figure 1).^39,54^ Dimethyl(methylthio)sulfonium trifluoromethanesulfonate (DMTST) served as a thiophilic promotor for all glycosylations,^41−43,55^ enabling the synthesis of a library of protected GXM oligosaccharides (**27**–**35**) (SI Schemes 1 and 2). Although recent efforts allowed the assembly of protected GXM glycans up to an 18-mer, problems were encountered in the deprotection (catalytic hydrogenolysis) of larger structures.^39^ Addressing this limitation, improvements were made regarding palladium on carbon (Pd/C) selectivity for hydrogenolysis and the identification of a quality Pd/C catalyst.^49,50^ Using these optimized hydrogenolysis conditions,^35,42^ the entire library of GXM glycans (**6**–**10**, **16**–**18**, **25**, and **26**) was successfully deprotected in good yields (70–90%), with careful pH control to ensure the stability of the 6-*O*-acetyl esters (SI Scheme 1).

**Figure 2:**
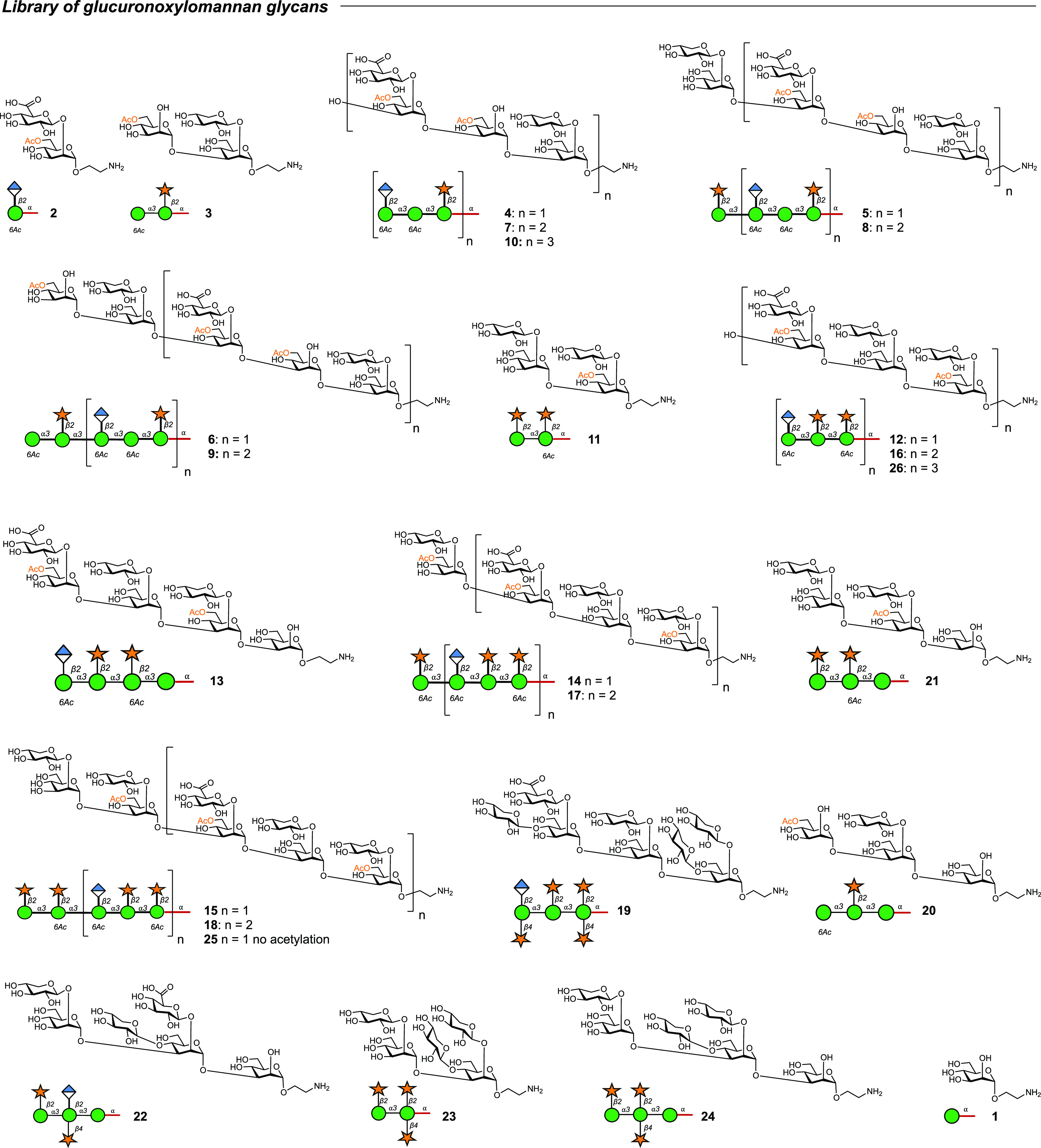
Library of synthetic glucuronoxylomannan glycans.

The 26 deprotected GXM structures were then printed using a non-contact microarray printer (Figure 2). Glycan microarrays offer an ideal platform for high-throughput analysis of glycan–protein interactions and have been effectively used to define lectin and antibody epitopes.^39,56−59^ A non-natural, synthetically installed amino linker present on the reducing end facilitated covalent attachment of the synthetic glycans to *N*-hydroxysuccinimide ester-functionalized glass surfaces. Each compound was printed in replicate (×5) and at the concentration of 200 μM.^39^

### Conserved Molecular Reactivities toward the M2 Motif GXM Is Serotype-Independent and Consistent across Infection and Vaccination

The binding specificity of 16 mAbs (Table 1) to the microarray was investigated. Immunization with GXM-TT (serotype A patient isolate strain NIH-371) conjugate vaccination of mice (BALB/c) resulted in the generation of B cell clones, from which mAbs were subsequently developed.^60,63^ Specifically, antibodies 2D10, 3E5, and 2H1 were derived from one B cell clone, while antibodies 12A1, 13F1, and 18B7 were obtained from another B cell clone.^60^ mAb 3E5 was further developed into a family of six isotype variants by isolating spontaneous class-switched hybridoma cells.^64^ mAbs 21D2 and 4H3 (with three isotypes generated from the parent IgG_3_ hybridoma) were generated from B cells obtained from *C. neoformans* serotype D-infected mice.^61^

**Table 1:** Monoclonal Antibodies to GXM Used in This Study

Name	Origin	Isotype(s)	Light chain: kappa (κ) or lambda (λ)	Classification^a^	GXM motif reactivity^b^	Ref
18B7	serotype A, GXM-TT conjugate	IgG_1_	κ	P	M1, M2, and M4	Mukherjee et al. 1993^60^
12A1	serotype A, GXM-TT conjugate	IgM	κ	P	M1, M2, and M4	Mukherjee et al. 1993^60^
2H1	serotype A, GXM-TT conjugate	IgG_1_	κ	P	M2 and M4	Mukherjee et al. 1993^60^
2D10	serotype A, GXM-TT conjugate	IgM	κ	P	M2	Mukherjee et al. 1993^60^
21D2	serotype D infection	IgM	κ	NP	M2 and M4	Casadevall et al. 1991^61^
10F10	serotype A, GXM-TT conjugate	IgG_1_	κ	P	M2 and M4	Mukherjee et al. 1993^60^
13F1	serotype A, GXM-TT conjugate	IgM	κ	NP	M2 and M4	Mukherjee et al. 1993^60^

3E5	serotype A, GXM-TT conjugate	IgG_1_	κ	P	M2 and M4	Mukherjee et al. 1993^60^
		IgG_2a_		P	M2 and M4	
		IgG_2b_		P	M2	Janda et al. 2015^62^
		IgG_3_		NP	M2	
		IgA		P	M2	
		IgE		P	M2	

4H3	serotype D infection	IgG_1_	λ	NP	M2	Casadevall et al. 1991^61^
		IgG_2b_		P	M2 and M4	
		IgG_3_		DE	M2	

a Protective (P), non-protective (NP), or disease-enhancing (DE).

b As defined by the GXM microarray.

**Figure 3:**
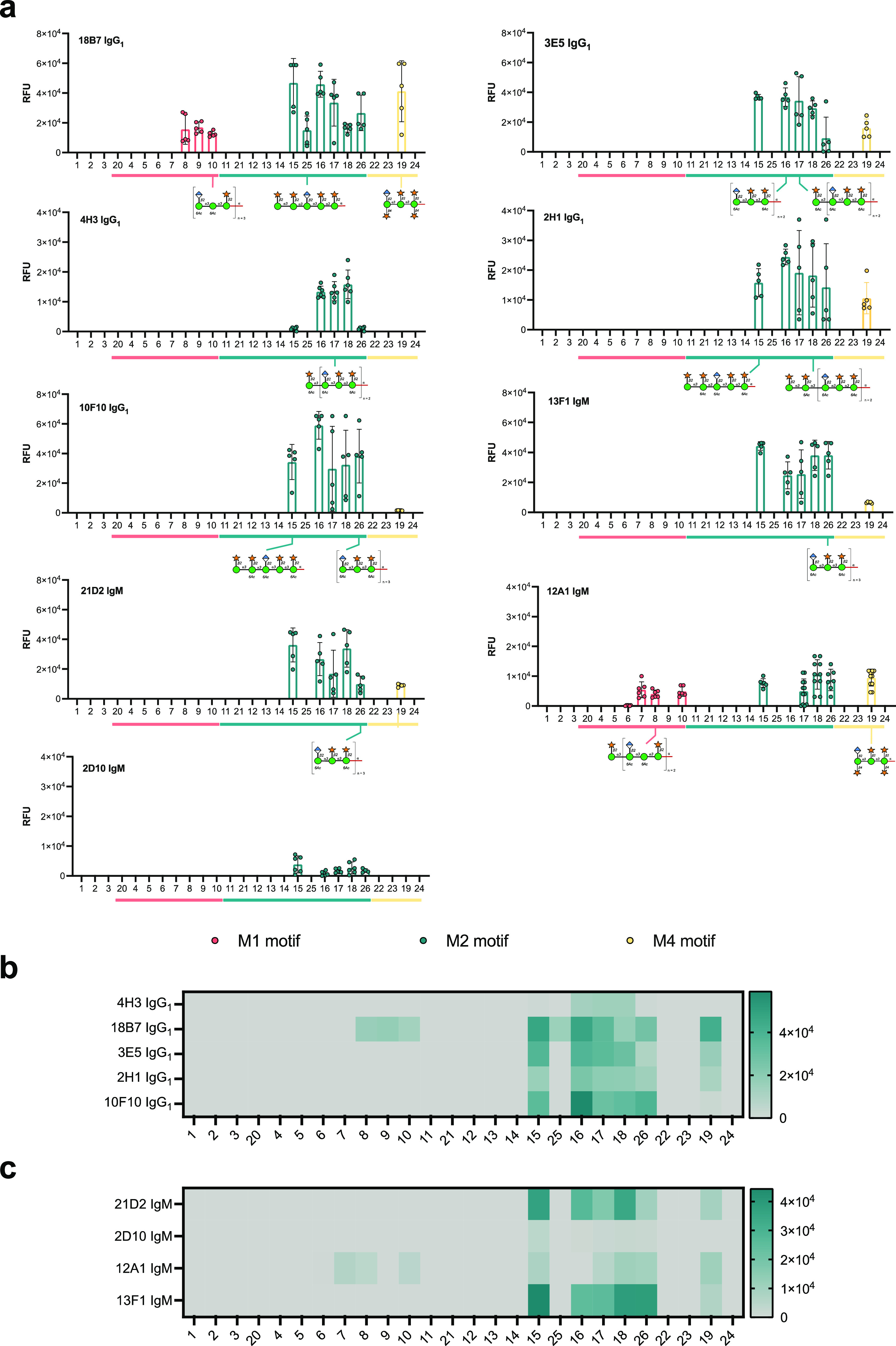
Microarray profile of several mAbs against synthetic GXM microarray. (a) Binding profile of seven mAbs. (b) Heatmap of IgGs binding to microarray. (c) Heatmap of IgMs binding to microarray. *X*-axes are glycan numbers defined in Figure 2.

**Figure 4:**
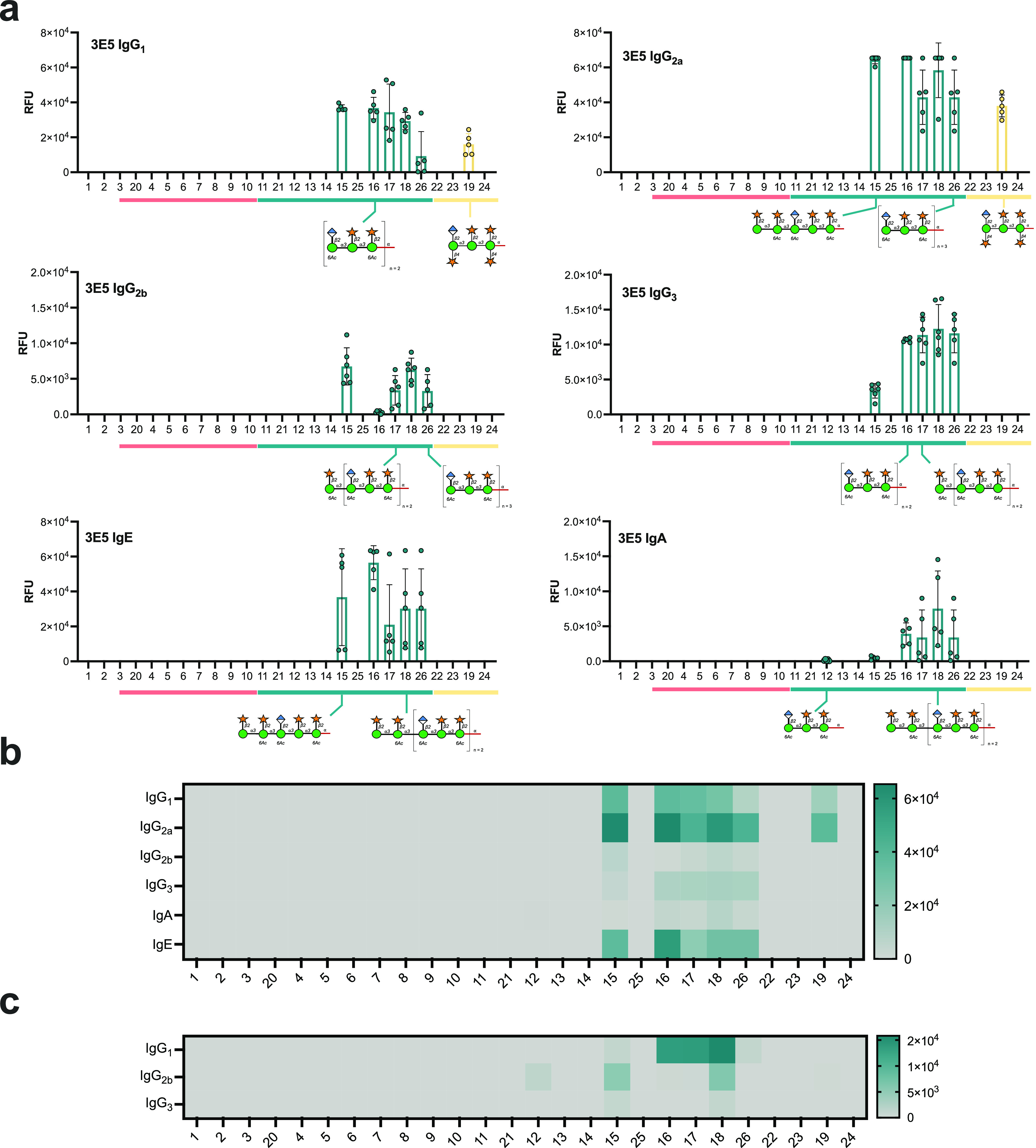
Isotype switching alters GXM antibody specificity and affinity. (a) Microarray binding data for mAbs 3E5 IgG_1_, 3E5 IgG_2a_, 3E5 IgG_2b_, 3E5 IgG_3_, 3E5 IgE, and 3E5 IgA. (b) Heatmap of mAb 3E5 isotype variants binding to microarray. (c) Heatmap of mAb 4H3 isotype (IgG_1_, IgG_2b_, and IgG_3_) variants binding to microarray. *X*-axis are glycan numbers defined in Figure 2.

Analysis of mAb binding revealed a few distinct observations. First, we identified conserved binding reactivity to the GXM M2 motif, independent of the origin or the function of the mAb (Figures 3 and 4, and SI Figure 2). All 16 mAbs tested bound M2 motif structures, and some mAbs displayed selectivity for the M2 motif, e.g., 2D10. In general, for antibodies to bind to the M2 motif, oligosaccharides larger than ≥10-mers were required. Increasing the sizes of the glycans beyond that of a 10-mer (M2 motif, **15**) or 12-mer (M1 motif, **8**) did not consistently lead to higher relative fluorescence units (RFUs). The minimal epitope shared among protective IgGs resembles short M2 motif-like glycans, composed of a pentasaccharide α-1,3-mannan (SI Figure 1). In contrast, the minimal epitope of IgGs not associated with protective efficacy was more extended, necessitating an octasaccharide of α-1,3-mannan for optimal binding to M2 motifs.

Additionally, a subset of the antibodies showed cross-reactivity. 12A1 and 18B7 demonstrated cross-reactivity to both M1 and M2 motif glycans, suggesting that, for these mAbs, binding to the central β-1,2 xylose branch of M2 motif glycans is non-essential (Figure 1). While nine mAbs (12A1, 18B7, 2H1, 21D2, 10F10 (weakly), 13F1, 3E5-IgG_1_ and 3E5-IgG_2a_ isotypes, and 4H3-IgG_2b_ isotype (weakly)) exhibited cross-reactivity to both M2 and M4 motif glycans (8-mer, **19**). Among non-protective IgGs, a trend was observed of low tolerance for β-1,4 branches (M4 motif) and a strict requirement for mannan backbone acetylation (SI Figure 1). The high degree of xylose branching of the M4 motif seems to eliminate the need for *O*-acetylation. This is in contrast to the M2 motif, where acetylation is often essential for antibody recognition. Two antibodies, mAbs 4H3 and 21D2, were raised in response to infection with a serotype D (M1 motif) strain, however, on the microarray they exhibited no reactivity toward M1 motif glycans, raising questions about the validity of the historical connection between serotype and polysaccharide structure.^65,66^

### Role of Xylose Branching in GXM Motif Binding

For binding to M1 and M2 motifs, larger α-mannan backbones (≥5 units) and 6-*O*-acetylation were generally necessary for detection by microarray.^34^ In contrast, the M4 motif (**19**), which is recognized by nine mAbs, contains only an α-mannan trisaccharide and lacks 6-*O*-acetyl esters but does contain a greater amount of xylose branching (Figure 1). An explanation for binding to M4 motifs can be rationalized through molecular modeling, which predicts that M4 glycans have more restricted backbone torsion angles and therefore a higher rigidity structure.^67^ This increased rigidity of the α-mannan backbone appears to allow for antibody–glycan interactions on smaller but more highly branched glycans. Binding to smaller glycans also containing [xylose-β-1,4]-branches, **22**, **23**, and **24**, was not observed. Glycan **22** contains the glucuronic acid β-1,2-[xylose-β-1,4]-mannoside unit, while glycans **23** and **24** contain the xylose β-1,2-[xylose-β-1,4]-mannoside branching patterns. As none of these glycans were recognized, it may suggest the cumulative effect of each saccharide is vital for the overall M4 motif conformation to be assumed.

### Cross-Reactivity to M2 and M4 GXM Motifs Is Associated with Positive Outcomes in Passive Administration

The most common cross-reactivity in this study was between the M2 and M4 motifs. IgG antibodies with differing efficacies were found to bind to the M2 motif (10-mer, **15**); however, protective antibodies showed higher RFUs compared to non-protective antibodies (Table 1 and SI Figure 2a,c). Notably, cross-reactivity to the M4 motif (8-mer, **19**) appeared to correlate with greater protective capacity in IgG mAbs, as only antibodies associated with protective outcomes (18B7, 2H1, 10F10, 3E5-IgG_1_, 3E5-IgG_2a_, and 3E5-IgG_2b_) showed this binding capacity, whereas the IgG antibodies associated with non-protective outcomes (3E5-IgG_3_, 4H3-IgG_1_ and -IgG_3_) did not exhibit cross-reactivity to the M4 motif (SI Figure 2b). This suggests that GXM motif cross-reactivity could be a key component of an efficacious IgG mAb in passive administration. However, we note that the same cross-motif reactivity trends are not true across other isotype classes, i.e., IgM, IgA, or IgE.

Overall, the microarray and molecular modeling data support the notion that smaller glycans cannot fully mimic the intricate secondary structure of GXM, providing an explanation for why a synthetic M2 motif heptasaccharide–human serum albumin conjugate was found to be non-protective *in vivo*.^38,68^ The microarray and molecular modeling data further suggest that, to effectively mimic the M2 motif secondary structure, a 10-mer glycan is minimally required (Figure 3).^69,70^

### Role of *O*-Acetylation in Antibody Binding to M2 Motif GXM

All antibodies required 6-*O*-acetylation for binding to M2 motif **15** with the exception of 18B7 (Figures 3 and 4, and SI Figure 2). The role of *O*-acetylation in antigen binding could be due to its inclusion as a component of an antibody epitope or due to its effects on the secondary structure of GXM.^34,63^ Comparing microarray and ELISA data requires caution, as ELISA plates utilize polysaccharide isolated from *C. neoformans* strains, which often contain multiple GXM motifs. This is in stark contrast to the microarray, where well-defined synthetic glycans with single GXM motifs are presented. Despite these differences, both methods suggest the importance of *O*-acetyl groups in antibody–glycan interactions. De-*O*-acetylated GXM in ELISA assays has been found to reduce or abolish binding by mAbs,^71^ and on the microarray, deacetylation of the M2 motif (10-mer **15** → 10-mer **25**) abolished binding for most mAbs (15 out of 16). The finding that mAb 2H1 was unable to bind non-acetylated glycan **25** complements previous findings that *O*-acetylation is important for its ability to bind to fungal cells and that non-acetylated *C. neoformans* strains had a greater ability to escape macrophages when mAb 2H1 mediated the phagocytosis.^72^^71^

### Broadly Protective mAbs 18B7 and 12A1 Exhibit Diverse Molecular Reactivity to Glucuronoxylomannan Motifs

mAbs 18B7 and 12A1 were generated from B cells recovered from a mouse immunized with a GXM-TT conjugate vaccine, and mAb 18B7 was tested in a phase I clinical trial.^60,63,73^ mAbs 18B7 and 12A1 exhibited distinct cross-reactivity for M1, M2 and M4 motif glycans (Figure 3). mAb 12A1 manifested no significant difference in binding affinity to the M1 motifs (10-mer, **7**) or M2 (10-mer, **15**) but did display greater affinity (**, *p* = 0.0092) for the M4 motif (8-mer, **19**) compared to that of the M1 motif (10-mer, **7**). When comparing mAbs 12A1 and 18B7 binding to M1 (**7**, 10-mer, M1 motif) and M2 (**15**, 10-mer, M2 motif) motif glycans the importance of the presence of the central xylose branch was evident as it enabled increased binding for mAb 12A1, while its absence abolished binding for mAb 18B7 (Figure 3a). A key difference between these mAbs is that 18B7 recognized the non-acetylated M2 motif (10-mer, **25**). Though, it should be noted that comparing reactivity between M1 and M2 motif glycans is challenging due to their differing 6-*O*-acetylation patterns. To elicit broad antibody binding reactivity from a vaccine, our results suggest that one strategy could involve creating a trivalent conjugate containing antigens from the M1 (10-mer, 7), M2 (10-mer, 15), and M4 (8-mer, 19) motifs. Such a formulation has the potential to generate a polyfunctional antibody response that could mimic the diverse molecular reactivity of mAbs 18B7 and 12A1.

### Isotype Switching Alters GXM Antibody Specificity and Affinity

The effector functions of mAbs mediate antibody interactions with cells of the innate immune system via the Fc region. How isotype class switching affects antibody–glycan interactions in the Fab is not well established. Here, we probed this in detail with a set of two antibodies (3E5 and 4H3) for which isotype variants had been generated.

mAb 3E5, developed using hybridoma technology, was diversified into a family of isotype variants.^64^ This family consisted of five protective isotype-switched antibodies (IgG_1_, IgG_2a_, IgG_2b_, IgE, and IgA) and the original non-protective IgG_3_. Isotype switching in the 3E5 mAbs induced changes in the paratope conformation, impacting glycan binding reactivity on the microarray (Figure 4a and b).^74^ Certain isotypes (IgG_2b_, IgG_3_, IgE, and IgA) showed specificity toward M2 motifs, while other isotypes (IgG_1_ and IgG_2a_) demonstrated cross-reactivity to M2 and M4 motifs. All members of the 3E5 antibody family required 6-*O*-acetylation to bind to the M2 motif glycans. Further, isotype switching affected binding affinity, with the weakest binding protective antibody (IgG_2b_) exhibiting higher affinity than the non-protective (IgG_3_) for the M2 motif (10-mer, **15**) (* p = 0.0151).

mAb 4H3 *in vivo* protective efficacy is ranked as IgG_2b_ > IgG_1_ ≫ IgG_3,_ with the latter being disease-enhancing.^64^ On the microarray, the isotype-switched variants (IgG_2b_ and IgG_3_) bound only to glycans **15** (10-mer) and **18** (16-mer) but not to the M2 motif repeats of **16** (12-mer) and **17** (14-mer), indicating a role for the terminal tetrasaccharide unit in binding (Figure 4c and SI Figure 3). However, these isotype variants did not bind to the smaller M2 motif (4-mer, **11**) that contains this epitope, implying that these smaller glycans have more dynamic structures that do not reflect larger oligosaccharides.^69,70^ The IgG_1_ isotype variant bound more strongly to glycans **15**–**18** than the IgG_2b_ variant, even though the latter has been found to provide better *in vivo* protection. This suggests that epitope binding strength of a mAb is not the sole determining factor in effective antibody protection. Additionally, mAb 4H3 IgG_2b_ binds stronger to **15** and to **12**, a shorter M2 motif glycan. Overall, the data here is consistent with other reports that isotype affects fine specificity but here the precise molecular consequences of isotype switching can be observed with molecular precision on the microarray.^75,76^

### Paratope Interactions with GXM Underscore the Importance of Glucuronic Acid

**Figure 5:**
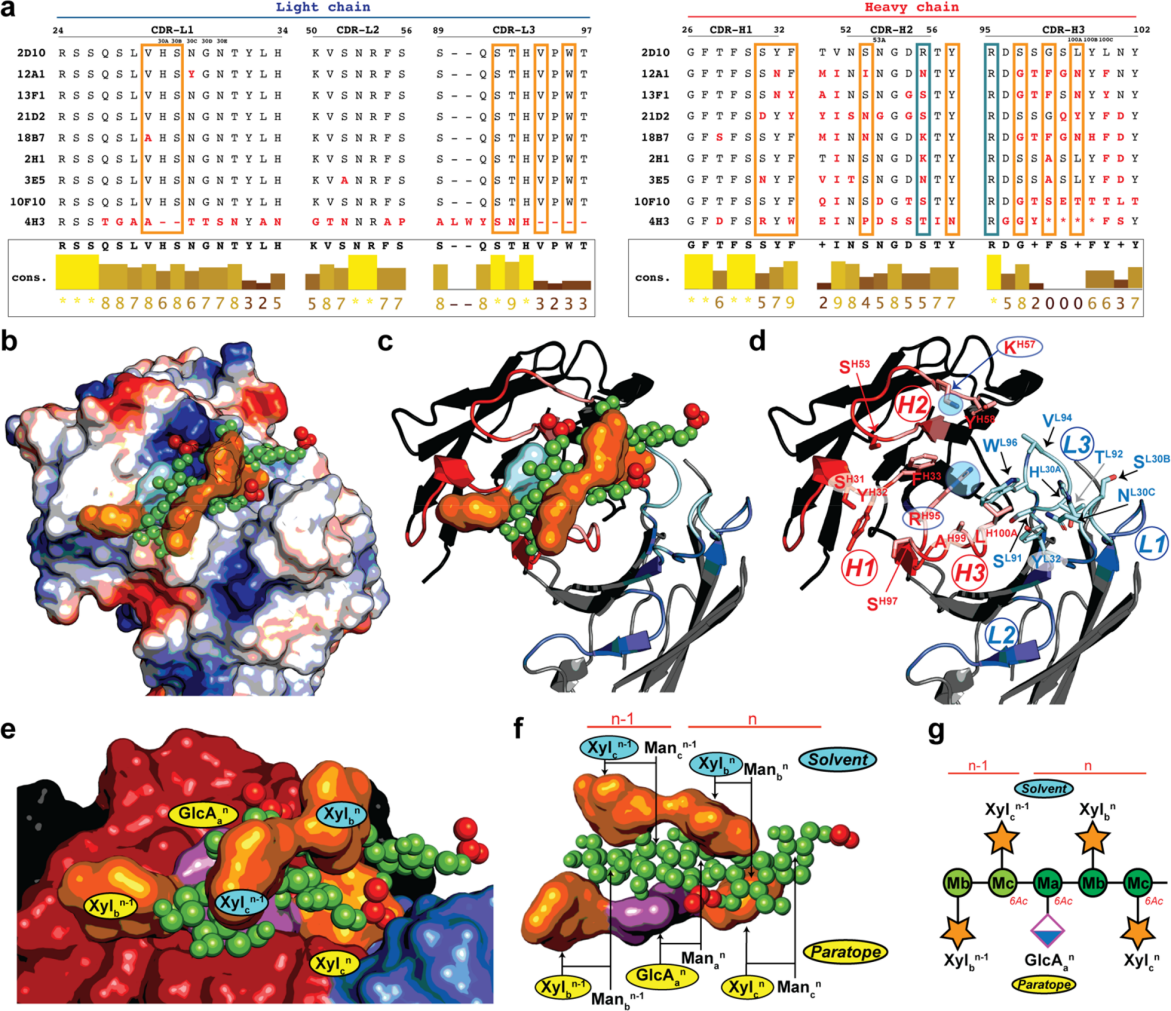
(a) Multiple sequence alignment analysis of complementarity-determining regions (CDRs) of GXM-specific mAbs. CDRs are numbered according to the Chothia numbering scheme. Positions highlighted with orange boxes make contacts with the GXM antigen in the putative model of the 2H1:GXM (M2 motif) 10-mer **15**. Positions highlighted in cyan boxes also make contact with the GXM antigen and are basic residues that contribute to the electropositive region of the paratope. (b) Representative model of the 2H1:GXM complex from Vina-Carb molecular docking, with the 2H1 Fab represented as an electrostatic surface. The GXM antigen is displayed with the mannan backbone and 6-*O*-acetylation as green and red spheres, and the xylose and glucuronic acid side chain glycans are displayed as orange and cyan surfaces. (c) The same model of the complex, with the 2H1 Fab displayed in cartoon representation and light- and heavy-chain CDRs highlighted in blue and red, respectively. (d) Expanded view of the interacting residues in this model, where contacting residues of mAb 2H1 are displayed as sticks and colored according to their CDR, as in panel c. The basic residues that form the electropositive pocket are highlighted with blue boxes. (e) Expanded view of the modeling results Motif 2 glycan docking the with mAb 2H1. Glycan side chains that are paratope- or solvent-facing are indicated with yellow and blue labels, respectively. (f) GXM **15** is extracted from the docking complex with 2H1 and rotated slightly to clearly demonstrate the solvent-exposed and paratope-interacting faces of the glycan, which are formed by alternating glycan branches along the mannan backbone. (g) Symbol Nomenclature for Glycans (SNFG) representation of **15**.

Mice exhibit a highly restricted immune response to GXM with regard to immunoglobulin V gene usage in response to both infection and vaccination. The majority of mAbs isolated with GXM-specificity use sequences from V_H_7183 and V_κ_5.1 germline gene elements,^60^ although a subset of mAbs produced from infection with *C. neoformans* (GH) use V_H_441 and V_λ_2 gene elements.^61^ Sequence analysis of complementarity-determining regions (CDRs) in all mAbs reveals a conserved electropositive region. This region includes an invariant arginine residue at position 95 (CDR-H3) and a less conserved lysine or arginine residue at position 56 (CDR-H2) (Figure 5a). These residues together form a basic pocket that appears to be an ideal site for coordination with glucuronic acid residues, which are conserved across all GXM motifs. Molecular modeling studies further support the significance of the glucuronic acid branch, as it consistently docks in close proximity to the electropositive region in the top-scoring structures (Figure 5b–f). None of the mAbs bound to xylomannan oligosaccharides on the microarray (**11**, **20**, **21**, **23**, or **24**), which emphasizes the importance of the glucuronic acid branch for antibody recognition.

Only mAbs 18B7 and 12A1 recognized the M1 motif, which is consistent with our molecular modeling. This suggests that the M2 glycan can dock with the glucuronic acid branch interacting with the paratope, while the two adjacent xylose branches (Man-b^*n*^ and Man-c^*n*-1^) face the solvent (Figure 5f). The proximal substituents (Man-c^*n*^ and Man-b^*n*-1^) of both triads interact again with the paratope, providing additional stabilization (Figure 5e,f). As a result, M2 epitopes are bound in the paratope through a critical electrostatic interaction with the glucuronic acid and two additional stabilizing interactions with xylose branches from adjoining triads (Figure 5e–g). However, the M1 motif glycans lack the xylose on Man-b^*n*-1^, thus eliminating one of the xylose branch interactions modeled for M2 glycans. Additionally, M1 motif glycans exhibit higher conformational flexibility and therefore a more dynamic secondary structure as a consequence of reduced xylose branching.^67,69^ mAbs 18B7 and 12A1 distinctly bound M1 motifs, and both possess a unique Phe99 in their CDR-H3 regions (Figure 5a and SI Figure 4a), providing a plausible structural explanation for M1 specificity. This phenylalanine in CDR-H3 could occupy the space vacated by the Man-b^*n*-1^ β-1,2-xylose branches in M1 motif glycans and provide additional stabilizing CH−π stacking interactions.^77,78^ These interactions could enhance binding to the M1 GXM motif by stabilizing the more flexible structure through additional contacts with acetyl groups on the mannan backbone.^79^ Conversely, Phe99 could exchange into an alternate conformation to accommodate M2 and M4 motifs (SI Figure 4a), emphasizing the importance of paratope plasticity in enabling broad recognition of GXM motifs.

All mAbs exhibiting M2 and M4 cross-reactivity had an aromatic residue, a phenylalanine or tyrosine, at position 100C of CDR-H3. For mAbs 2D10 and 10F10, which do not exhibit strong M4 binding, this position was substituted with a leucine and threonine, respectively, suggesting an explanation as to why mAb 10F10 exhibits the weakest binding among all protective IgG_1_ mAbs. A phenylalanine exists in the alignment at the position for mAb 4H3 but the CDR-H3 is significantly shorter and the precise arrangement of that residue is likely not comparable to the other mAbs. The effect of this conserved aromatic residue (100C) on epitope specificity is likely indirect due to its distance from the binding site (SI Figure 4b). However, the presence of this bulky hydrophobic group at the interface of the V_H_ and V_L_ domains could have unknown implications for the orientation of the two specificity determining domains and overall paratope architecture.^80−82^ In the case of mAbs 4H3 and 3E5, the binding to M4 epitopes is dependent on the specific constant domains, suggesting that Fc modulation can influence the structure of the variable domain, and this can modulate the ability to bind to M4 epitopes.

### Leveraging Antibodies to Map the Antigenic Diversity of Cryptococcal Cells

**Figure 6:**
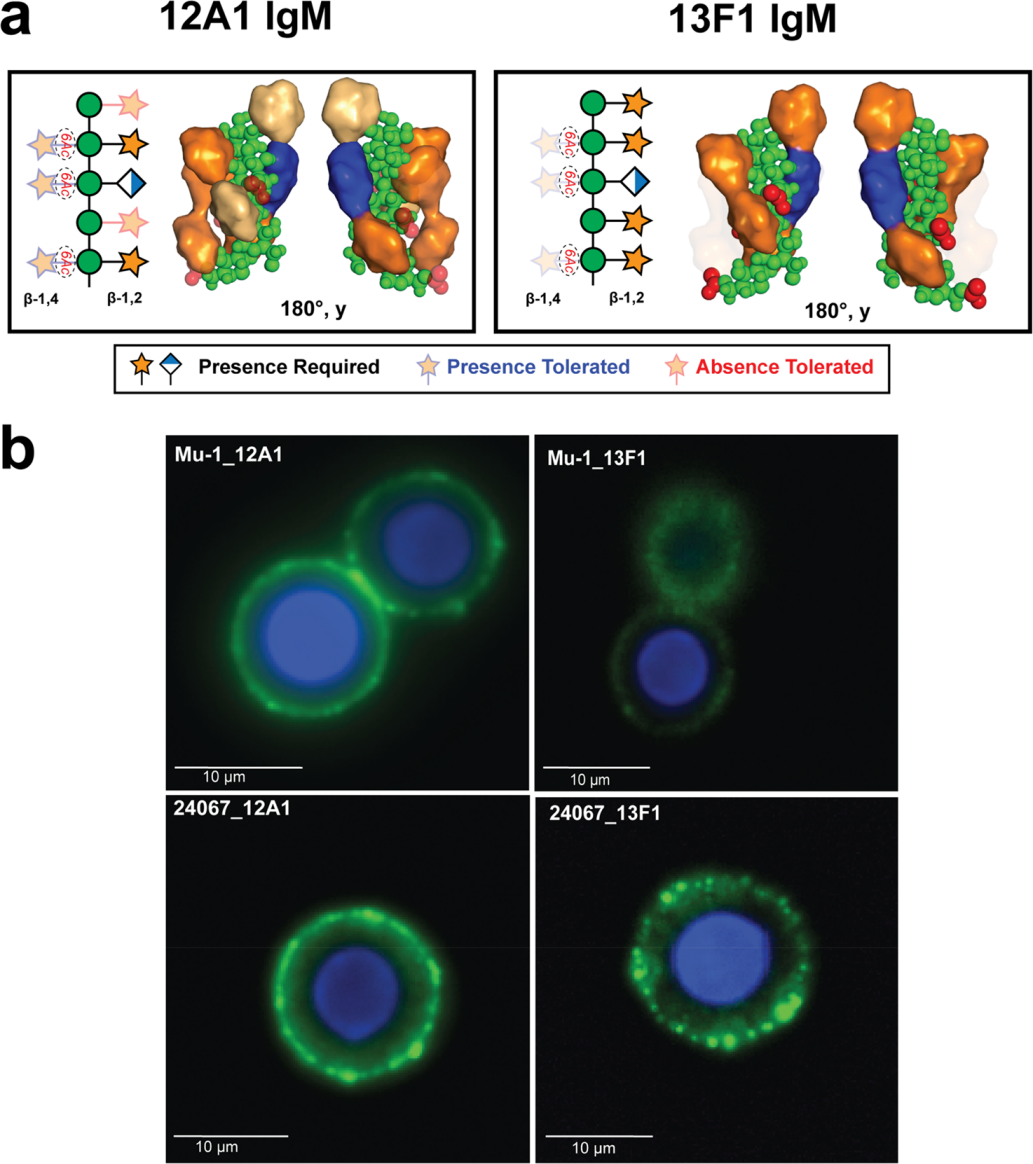
Composite epitopes recognized by mAbs 12A1 and 13F1 and antibody localization on capsules of single-motif *C. neoformans* strains. (a) Full reactivity profiles of mAbs 12A1 and 13F1. Each individual glycan in the array for which binding was observed is displayed with transparency levels that reflect the relative degree of binding, and the glycan structures are grouped into boxes with respect to their motif composition: M1 (red), M2 (green), and M4 (yellow). Energy-minimized models of the composite epitopes-based reactivity with glycan structures in the array. The mannan backbone and 6-*O*-acetyl groups are displayed as green or red spheres, respectively. Xylose or glucuronic acid branches are displayed as space-filling surfaces and are colored orange and blue, respectively. β-1,4-Xylose groups are displayed with a degree of transparency that reflects their relative binding to M4 structures compared to M2. The minimal glycan length was defined as the structure that was bound with the highest affinity in the array for a given mAb. For 12A1, xylose groups on mannose-b^*n* and *n*-1^ are colored a slightly lighter shade of orange to reflect the tolerance to the absence of these groups for these mAbs. (b) Immunofluorescence staining with mAbs 12A1 and 13F1 on a single motif expressing *C. neoformans* cells. Left panels: Immunofluorescence staining of Mu-1 (M2) and ATCC 24067 (M1) *C. neoformans* with mAb 12A1. Staining is annular for both strains. Right panels: Immunofluorescence staining of Mu-1 and ATCC 24067 was performed with mAb 13F1. Mu-1 staining is annular, and ATCC 24067 staining is punctate.

Among the 16 mAbs studied, two closely related IgMs emerged as strong candidates for mapping the antigenic compositions of different *C. neoformans* strains. These mAbs, 12A1 and 13F1, shared a common B-cell lineage but exhibited distinct epitope specificities due to somatic mutations.^36,83^ These 12 amino acids changes result in substantial changes in their reactivity to GXM motifs on the microarray (Figure 3). The 13F1 mAb readily binds M2 motif glycans and weakly binds to the M4 motif glycan (Figure 6a). Although the changes weaken the overall binding affinity of mAb 12A1 for glycans, it led to a remarkable expansion of its GXM motif reactivity profile to include M1, M2, and M4 motifs. Comparing the binding intensities of the two mAbs toward M2 and M4 motifs revealed that mAb 12A1 bound with greater affinity to M4 motifs (**19**, *p < 0.0334), while mAb 13F1 exhibited much higher binding intensity toward M2 motifs (**15**, ****p ≪ 0.0001). This divergence in GXM specificity offers an explanation as to why 12A1 was found to be protective, while mAb 13F1 was non-protective in the context of a lethal infection of *C. neoformans* (ATCC 24067, serotype D, M1 motif).^83^

Previous research indicates that ATCC 24067 expresses only the M1 motif; however, microheterogeneity or microevolution of GXM has been documented to occur over time in laboratory culture.^84^ Despite this knowledge, the precise molecular details of this microevolution remain unknown. To address this, we conducted an exploration of the binding patterns of mAbs 12A1 and 13F1 on *C. neoformans* encapsulated cells, specifically ATCC 24067 (M1 motif) and Mu-1 (M2 motif), using immunofluorescence microscopy (Figure 6b).^15^ As anticipated, mAb 12A1 exhibited an annular binding pattern on both Mu-1 and ATCC 24067 fungal cells likely due to its broad GXM reactivity (Figure 6). Similarly, mAb 13F1 displayed an annular binding pattern on strain Mu-1, consistent with its ability to target M2 motifs. However, for strain ATCC 24067, mAb 13F1 demonstrated a punctate binding pattern, suggesting the presence of epitopes, namely M2 or M4 motifs. This observed partial binding aligns with the documented microheterogeneity in the ATCC 24067 capsule. This data expands our understanding to define that this microevolution involves the emergence of M2 and/or M4 motifs in ATCC 24067.^84^

We propose that the spatial distribution and density of antibody binding to the cryptococcal capsule play a pivotal role in determining the protective capabilities of antibodies. This binding is influenced by their epitope preferences and supports the case for employing mAbs with diverse GXM motif cross-reactivity for passive administration. A prophylactic vaccine should focus on eliciting a spectrum of antibodies capable of binding to the diverse chemical architecture of these pathogenic fungal cells.

## Conclusion

The data presented in this study challenges the traditional classification of antibodies and their respective epitopes as either “protective” or “non-protective”. The interactions of mAbs 18B7 and 12A1 with the glycan array emphasize the advantages of broad binding flexibility. While, the impact of isotype switching is demonstrated by families 3E5 and 4H3, where it was found to affect glycan binding and lead to the emergence of motif cross-reactivity. Two closely related mAbs, 12A1 and 13F1, with just a 12-amino-acid difference, showcase the remarkable precision of antibody fine specificity. These amino acids are the determinants that distinguish between the high-affinity, yet non-protective 13F1 and the lower-affinity, yet highly cross-reactive, and protective 12A1. Collectively, these findings underscore the context-dependent nature of the antibody function. Furthermore, the binding specificities of these antibodies were used to define the previously unknown source of heterogeneity within the ATCC 24067 strain to be the emergence of M2 or M4 motifs. The potency of an antibody against *C. neoformans* is not solely guided by its Fab and constant regions but is intricately linked to the epitope’s presence, distribution and abundance within the capsule. This more comprehensive understanding of the factors shaping the “protective” role of antibodies is important for advancing cryptococcal vaccine development. An outcome of this study is that it strongly suggests the need for a multivalent approach to designing glycoconjugate vaccines targeting *C. neoformans*. This multivalent approach is likely essential to evoke a strong and long-lasting antibody response.

## Methods

### General Notes

Silica gel flash chromatography was carried out using an automated flash chromatography system, Buchi Reveleris X2 (UV 200–500 nm and ELSD detection, Reveleris silica cartridges 40 μm, Büchi Labortechnik AG). Size-exclusion chromatography was performed on Bio-Gel P-2 (Bio-Rad Laboratories Inc.) using isocratic elution (H_2_O:*tert*-butyl alcohol, 99:1, v/v). Instrumentation: peristaltic pump P-3 (Pharmacia Fine Chemicals), refractive index detector Iota 2 (Precision Instruments), and PrepFC fraction collector (Gilson Inc.). Software: Trilution LC (version 1.4, Gilson Inc.). All chemicals for the synthesis were purchased from commercial suppliers (Acros, Carbosynth Ltd., Fisher Scientific Ltd., A/S, Merck, Sigma-Aldrich, VWR, Strem Chemicals, and AlfaAesar) and used without purification. Dry solvents were obtained from a PureSolv-EN solvent purification system (Innovative Technology Inc.). All other anhydrous solvents were used as purchased from Sigma-Aldrich in AcroSeal bottles.

### General Procedure for Catalyst Pretreatment^49^

500 mg of Pd/C (any commercial catalyst) was suspended in 1 mL of a dimethylformamide (DMF):H_2_O mixture (80:20 v/v), and the solution was made acidic by the addition of 200 μL of HCl (ACS Reagent, 37%, pH 2–3), with or without an atmosphere of hydrogen gas for about 20 min. The presence of dimethylamine was confirmed via ninhydrin staining. The treated Pd/C catalysts were re-isolated though filtration. The moistened catalyst was then used directly in the hydrogenolysis reaction.

### General Procedure for Hydrogenolysis Reaction^49^

The treated catalyst (0.2–0.5 molar equiv of palladium per benzyl group) was added to a solution of oligosaccharide (1 equiv) dissolved in tetrahydrofuran (THF):*tert*-butyl alcohol:phosphate buffered saline (PBS) (100 mM, pH 4) (60:10:30, v/v/v). The reaction was placed in a high pressure reactor at 10 bar and was monitored via normal phase TLC (acetonitrile:H_2_O mixtures) and MALDI-TOF mass spectrometry Once complete the reaction mixture was filtered through a plug of Celite and then concentrated *in vacuo*. The residue was then re-dissolved in a minimal amount of sterile water and purified with a Bio-Gel P2 column, after lyophilization to yield the desired product.

### Microarray Screening

Glycan array scanning followed published procedures.^85^ Primary mAbs to GXM or control Abs were prepared from stocks to the necessary concentration in 3% BSA in PBS-T. Biotinylated goat anti-mouse kappa chain or lambda chain Abs were used as secondary reagents for all primary antibodies. Detection was performed with the streptavidin-conjugated SureLight P3 fluorophore (Cayman Chemical Company, Ann Arbor, MI) at 5 μg/mL in PBS-T. Scanning was performed first with the primary Ab, then the secondary Ab, and then the fluorophore, with washes between each step. All hybridization steps were performed using the Agilent 8-well gasket system in a humidity-controlled rotating hybridization oven at 26 °C for 1–2 h. Washes (×3) in TRIS-buffered saline (pH 7.6, 0.1% Tween 20) (TBS-T) for 3 min and once for 3 min in TBS. Scanning was performed in an Agilent SureScan Dx microarray scanner with red wavelength emission detection. The data were processed on Mapix software. The mean fluorescent intensities (corrected for mean background) and standard deviations (SD) were calculated (*n* = 6). Data were fitted by using Prism software (GraphPad Software, Inc.). Bar graphs represent the mean ± SD for each compound.

### Molecular Modeling

GLYCAM-Web Carbohydrate Builder (https://glycam.org/cb) was used to construct energy-minimized models of structure **15** (M2 motif). Docking of this ligand to mAb 2H1 was performed with Autodock Vina, and this can also be performed at https://glycam.org/cb.^86−88^ Starting coordinates of the antibody were obtained from the crystal structure of 2H1 Fab (PDB ID: 2H1P). Targeted docking of the energy-minimized model of structure **15** to the 2H1 paratope was carried out by transforming the CDRs of the antibody to the center of the search box (grid scale = 30 Å × 30 Å × 30 Å, 0.375 Å spacing between grid points) during the AutoDock Vina docking runs. Twenty binding modes were output per docking run. The lowest energy interaction was selected as the top model and depicted in figures. PyMOL was used for visualization and figure creation.

### Monoclonal Antibody Preparation

3E5-IgG_3_ and 3E5-IgG_1_ were described previously.^89,90^ mAb 18B7 and 2H1, both IgG_1_, were obtained as previously described.^91,92^ The murine mAbs were purified by protein A or G affinity chromatography (Pierce) from hybridoma cell culture supernatants, concentrated, and buffer exchanged against 0.1 M Tris-HCl pH 7.4. mAb concentration was determined by OD_280_ measurement.

### Antibody Sequencing Analysis

Available amino acid sequencing data for VL and VH domains of GXM-specific mAbs were aligned using the T-Coffee multiple sequence alignment package with default settings in Jalview.^93^ Conservation scores and alignment figures were generated in Jalview.^93^

### Immunofluorescence Staining

Immunofluorescence was performed as previously reported.^94^ Briefly, cells were grown in Yeast Extract Peptone Dextrose (YPD) for 48 h at 30 °C, shaking. A 1:50 dilution from YPD culture was induced for capsule expression in minimal media for 3 days, at 30 °C shaking. Cultures were centrifuged at 10000*g* for 5 min to isolate cells. Cells were incubated with either 12A1 or 13F1 overnight, washed, and incubated with anti-IgM-FITC secondary antibody overnight, washed, and mounted on slides with Prolong Gold mounting media. Images were collected on Leica THUNDER Live Cell and 3D Confocal Microscope and Olympus IX 70 microscope (Olympus America, Melville, NY) with 60× numerical aperture 1.4 optics equipped with standard FITC and 4′,6-diamidino-2-phenylindole (DAPI) filters.

## Supporting Information Available

Supporting figures and compound characterization data (PDF)
