# Supplementary material for: Synthetic Glycans Reveal Determinants of Antibody Functional Efficacy against a Fungal Pathogen
Source: ACS Infect Dis. 2023 Oct 19;10(2):475–88. doi: 10.1021/acsinfecdis.3c00447 (PMC10862557; doi:10.1021/acsinfecdis.3c00447)
Supplement: Supplementary file 1 — id3c00447_si_001.pdf [file id3c00447_si_001.pdf]

Supporting Information

Synthetic glycans reveal determinants of antibody functional efficacy against a fungal pathogen

Conor J. Crawford,[1,2,3] Lorenzo Guazzelli,[1,4#] Scott A. McConnell,[2#] Orla McCabe,[1] Clotilde d'Errico,[1] Seth D. Greengo,[2] Maggie P. Wear,[2] Anne E. Jedlicka,[2] Arturo Casadevall,[2*] Stefan Oscarson[1*]

[1]Centre for Synthesis and Chemical Biology, University College Dublin, Belfield, Dublin 4, Ireland, D04 V1W8. [2]Department of Molecular Microbiology and Immunology, Johns Hopkins Bloomberg School of Public Health 615 North Wolfe Street, Baltimore, MD 21205, USA. [3]Current address: Max Planck Institute for Colloids and Interfaces, Am Mühlenberg 1, 14476 Potsdam, Germany. [4]Current address: Department of Pharmacy, Università di Pisa, Via Bonanno 6, 56126, Pisa, Italy.

[#]Equal contribution. *Equal contribution and corresponding authors. Emails: acasade1@jhu.edu stefan.oscarson@ucd.ie

Table of Contents

Supporting figures S3

Instrumental details S10

Compound characterisation S11

NMR spectra S21

Supporting Figures

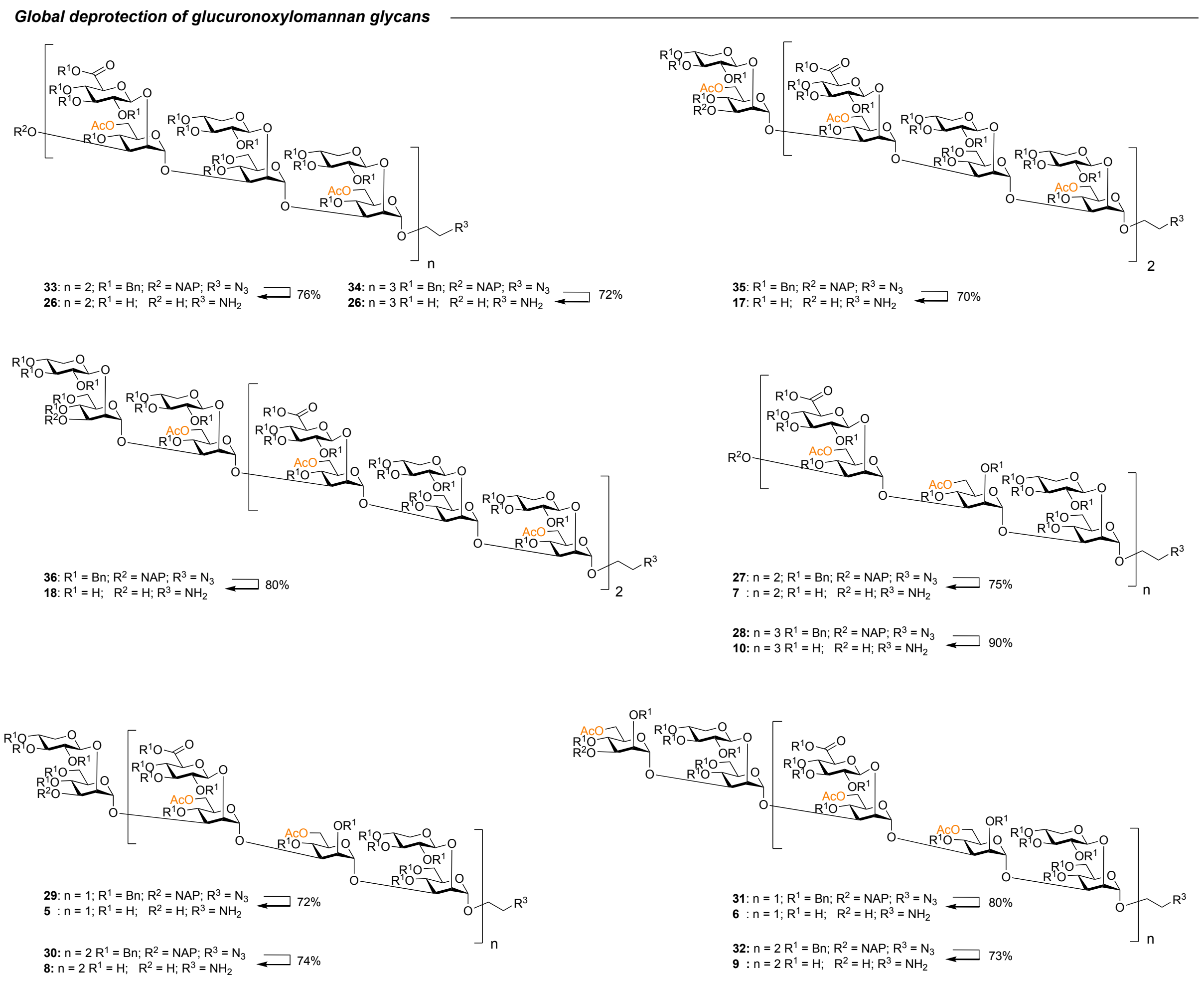

SI Scheme 1. Reagents and conditions: pre-treated 5 % Pd/C,[49] H_2(10 bar), THF:*t*BuOH:PBS (100 mM,pH 5) (60:10:30 v/v/v).

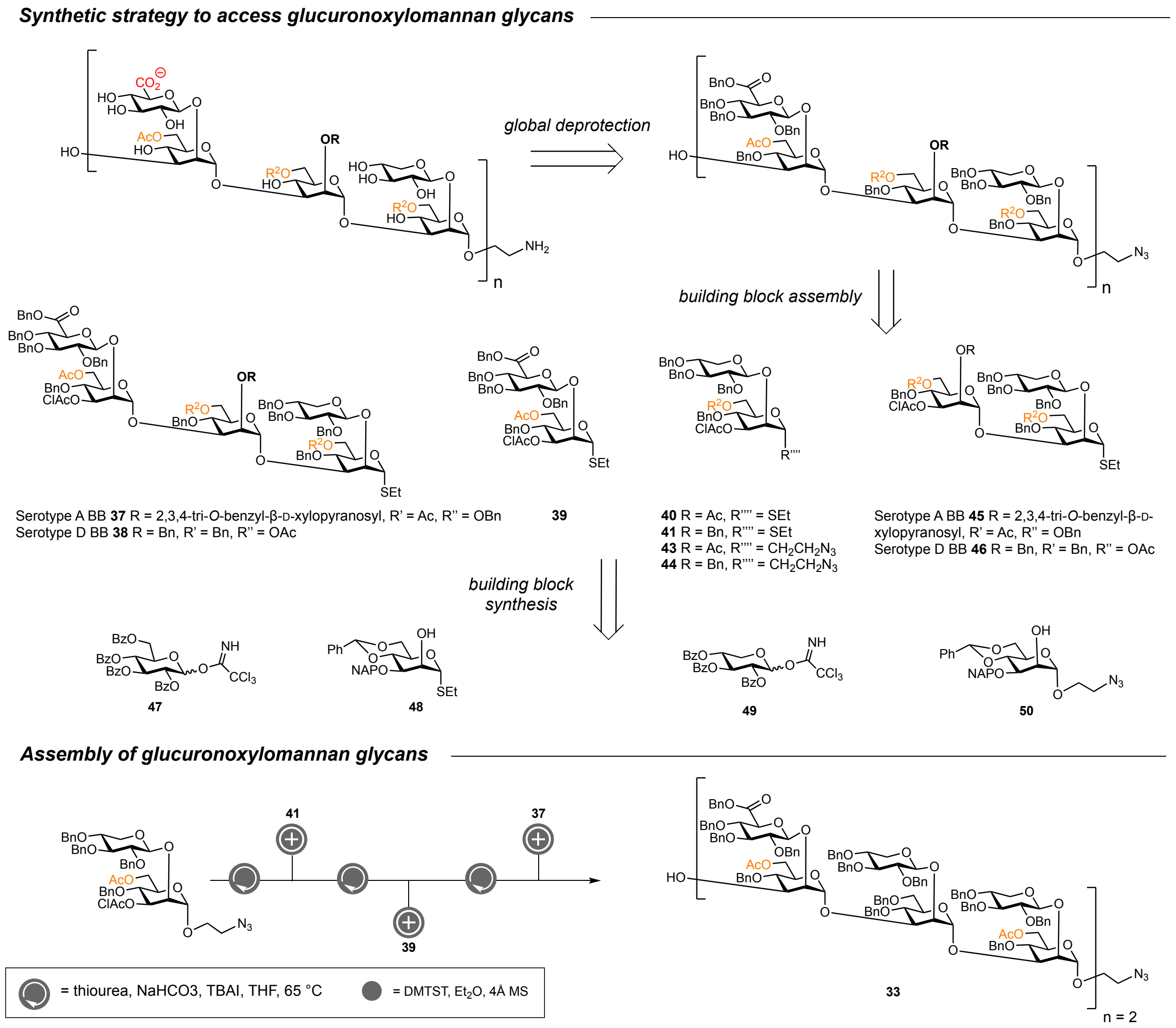

SI Scheme 2. Retrosynthesis of M1 and M2 motifs and assembly process towards glucuronoxylomannan glycans.

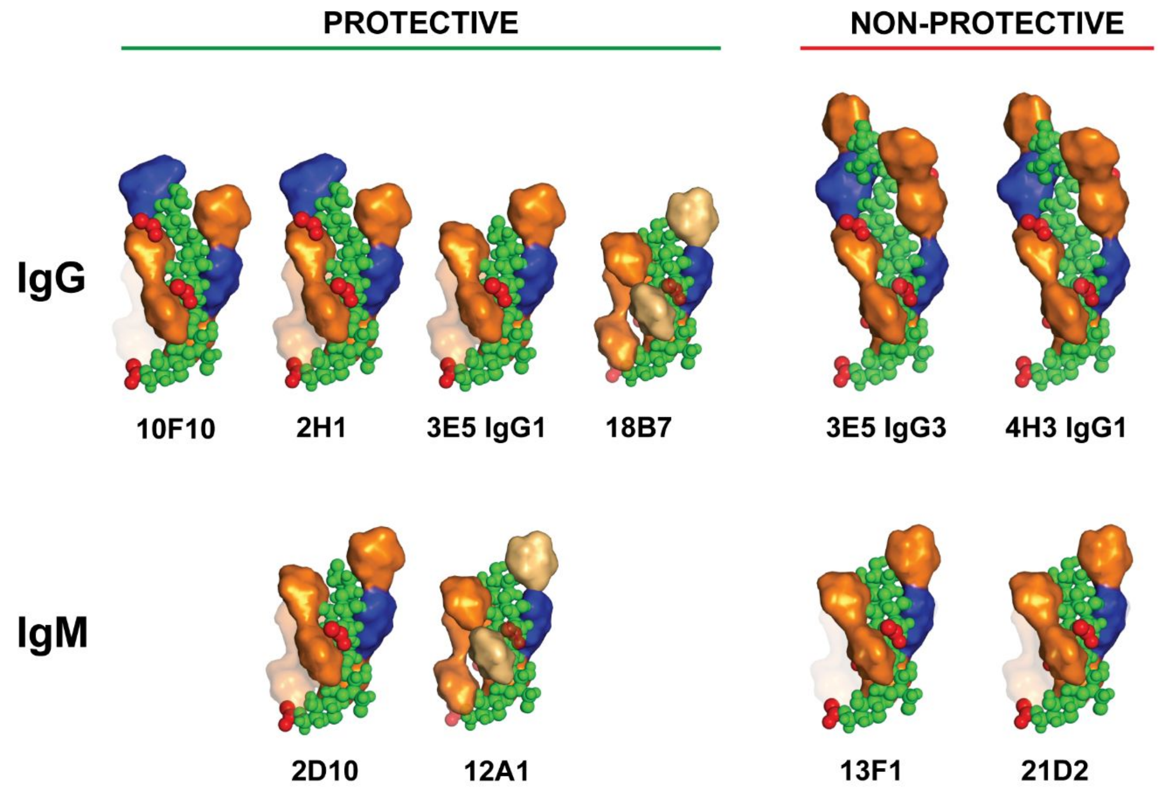

SI Figure 1. Composite epitopes recognized by protective and nonprotective mAbs in the array. a Energy-minimized models of the composite epitopes of each mAb tested based reactivity with glycan structures in the array. The mannan backbone and 6-*O*-acetyl groups are displayed as green or red spheres, respectively. Sidechain xylose or glucuronic acid residues are displayed as space-filling surfaces and are coloured orange and blue respectively. β-1,4 xylose groups are displayed with a degree of transparency that reflects their relative binding to M4 structures compared to M2. The minimal glycan length was defined as the structure that was bound with highest affinity in the array for a given mAb. For mAbs 12A1 and 18B7, xylose groups on mannose-b$^{n \text{ and } n-1}$ of the mannotriose repeat are coloured a slightly lighter shade of orange to reflect the tolerance to the absence of these groups for these mAbs.

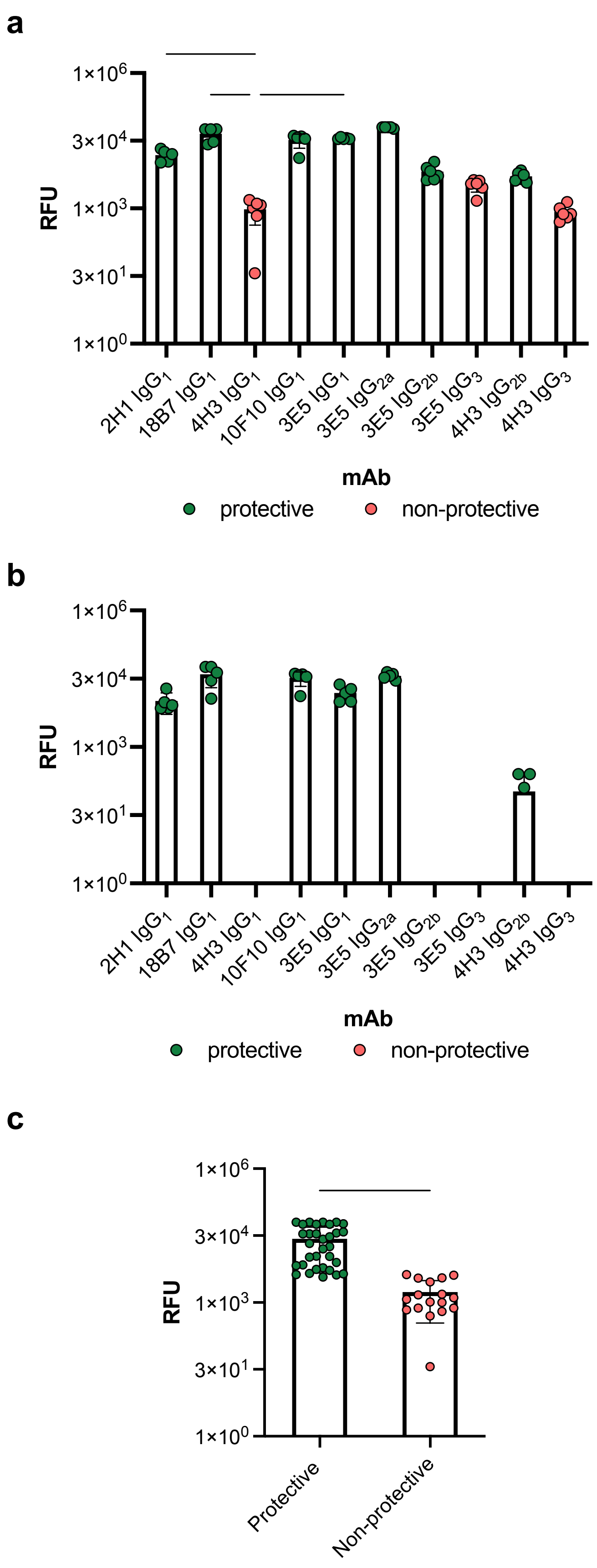

SI Figure 2. Comparing binding intensity of mAbs to M2 and M4 GXM motifs. a IgG_1 antibodies with differing efficacy were found to bind to the M2 motif (10-mer, **15**).

b Binding to the M4 motif (8-mer, **19**) appears to correlate with protective efficacy, as only protective IgG_1 antibodies (18B7, 2H1, 10F10, and 3E5) showed binding, while the non-protective IgG_1 mAb 4H3 did not exhibit binding to the M4 motif. **c** protective antibodies show higher relative fluorescence units (RFUs) compared to non-protective antibodies to M2 motif (**15**)

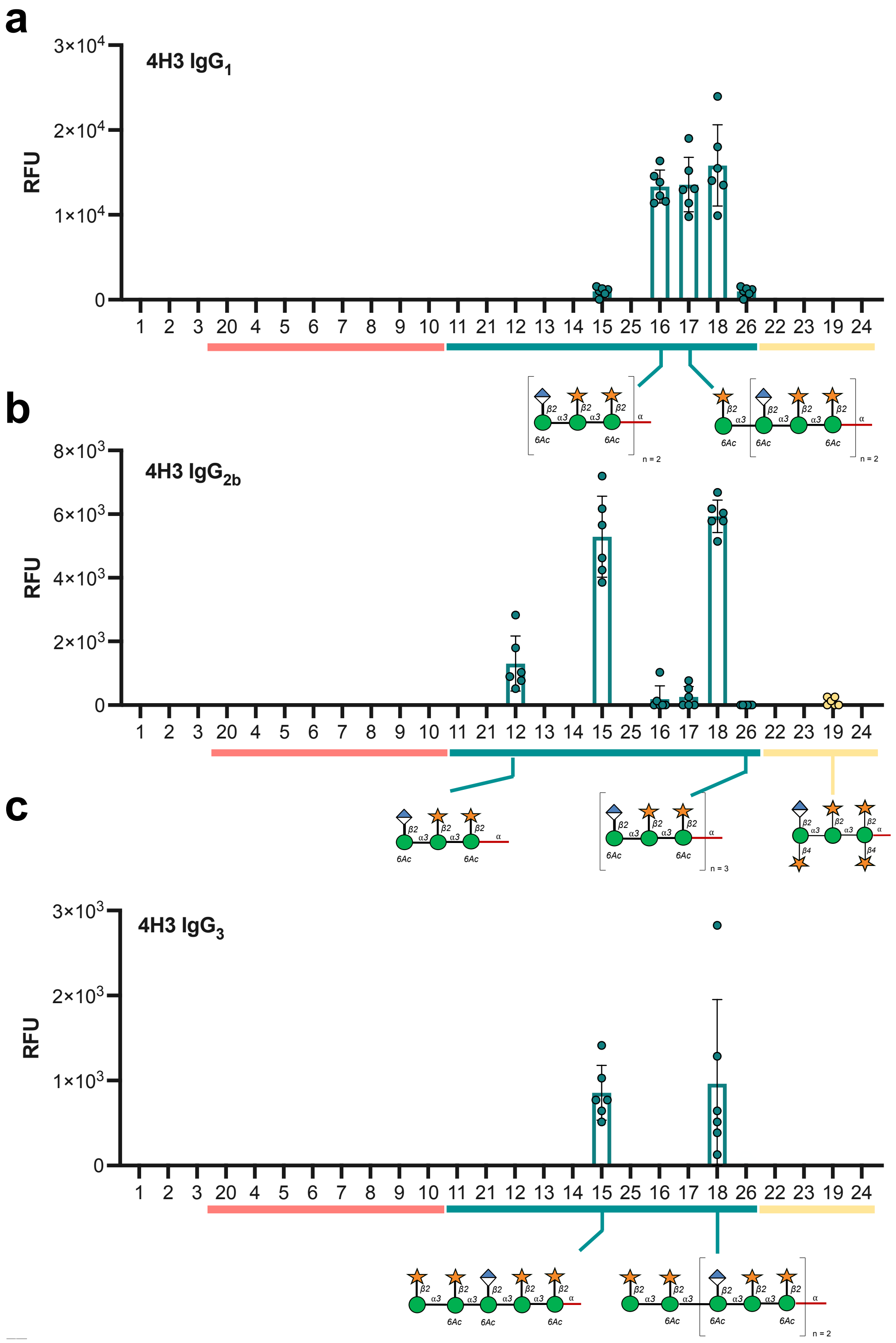

SI Figure 3. Mapping the effect of isotype switching on mAb 4H3. a microarray binding data for 4H3 IgG_1. **b** microarray binding data for 4H3 IgG_{2b}. **c** microarray binding data for 4H3 IgG_3. **d** heatmap of mAb 4H3 isotype variants binding to microarray**.**

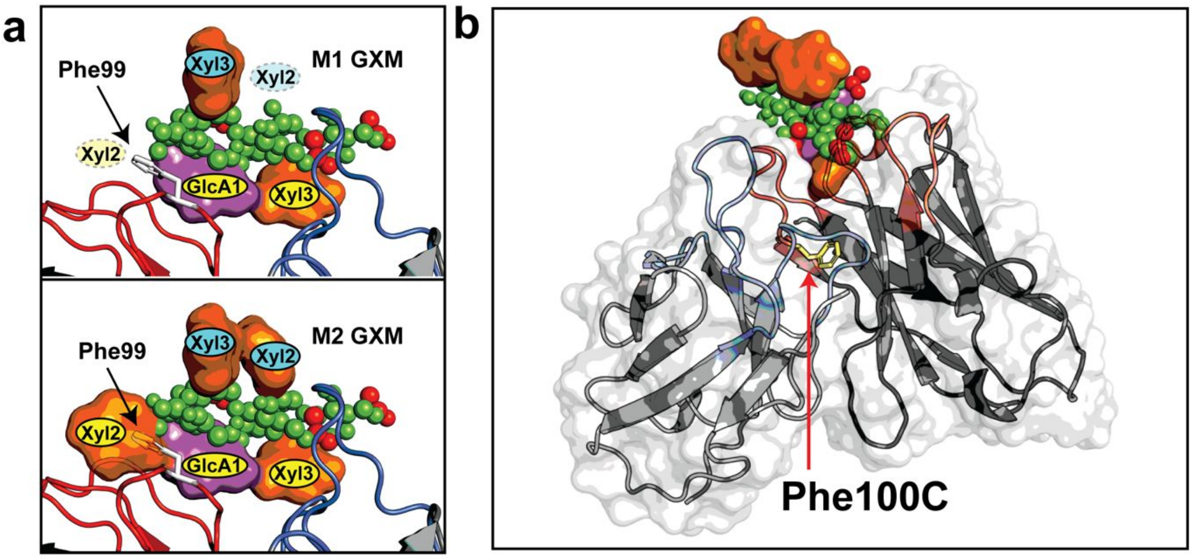

SI Figure 4. Paratope interactions with different GXM glycan motifs. a An expanded view of the top scoring model of 15 (Motif 2) glycan with the crystal structure of mAb 2H1 (PBDID: 2H1P). mAb 2H1 variable domains are visualized in surface representation to show the paratope surface. CDRs contributed by the heavy and light chains are coloured red and blue, respectively. The mannan backbone of **15** and 6-*O*-acetylations are displayed as green and red spheres. Xylose and glucuronic acid branches are shown in surface representation and coloured orange and purple, respectively. Glycan side chains that are paratope- or solvent-facing are labelled with yellow and blue labels, respectively. The position of Phe99, conserved in M1-binding mAbs 18B7 and 12A1, is demonstrated in the context of the complex between mAb 2H1 and 15. *Top panel:* Phe99 could fit into the volume vacated by the missing Xylose-2 on the (n-1) triad (dashed yellow label) and provide additional stabilizing interactions to Motif 1 glycans. *Bottom panel:* Most M2-binding mAbs have a small residue at this position to accommodate the volume of Xylose-2. Binding of Motif 2 GXM by 18B7 or 12A1 would require Phe99 to flip out to a different conformation, as there is a steric clash with Xyl2 of the (n-1) triad. **b** Phe100C (CDR-H3) is not part of the the predicted epitope docking site. However, this bulky residue is located at the interface between variable domains and could modulate the relative orientation of the domains, thus remodelling the paratope and altering specificity.

Instrumental Details

^1H NMR (400, 500, or 600 MHz) and ^{13}C NMR (101 MHZ or 125 MHz) spectra were recorded on Varian-Inova at 25 °C in water-d2 (D_2O), and ^1H NMR spectra were standardized against the residual solvent peak (D_2O, δ = 4.79 ppm; d6-DSS δ = 0.0 ppm). All NMR data are represented as follows: chemical shift (δ ppm) and multiplicity (s = singlet; d = doublet; t = triplet; q = quartet; dd = doublet of doublets; ddd = doublet of doublets of doublets; dt = doublet of triplets; m = multiplet; br = broad signal; ad = apparent doublet; and at = apparent triplet), coupling constant in Hz, integration. Assignments were aided by homonuclear 1H-1H (COSY, TOCSY, and ^1H-^{13}C heteronuclear (HSQC, HMBC) two-dimensional correlation spectroscopies. ^{13}C chemical shifts were reported with one digit after the decimal point, unless an additional digit was reported to distinguish overlapping peaks. Software used for data processing was MestReNova, version 11.0.0–17609 (MestReLab Research S.L.). High-resolution MS data were recorded on a Waters micromass LCT LC-Tof instrument using electrospray ionization (ESI) in either positive or negative mode. Low-resolution MS experiments were recorded on a Waters micromass Quattro Micro LC-MS/MS instrument using ESI in either positive or negative mode.

Compound Characterisation

2-Aminoethyl β-D-xylopyranosyl-(1→2)-α-D-mannopyranoside-[β-D-glucopyranosyluronic acid-(1→2)]-6-*O*-acetyl-α-D-mannopyranosyl-(1→3)-6-*O*-acetyl-α-D-mannopyranosyl-(1→3)-[β-D-xylopyranosyl-(1→2)]-α-D-mannopyranoside (5)

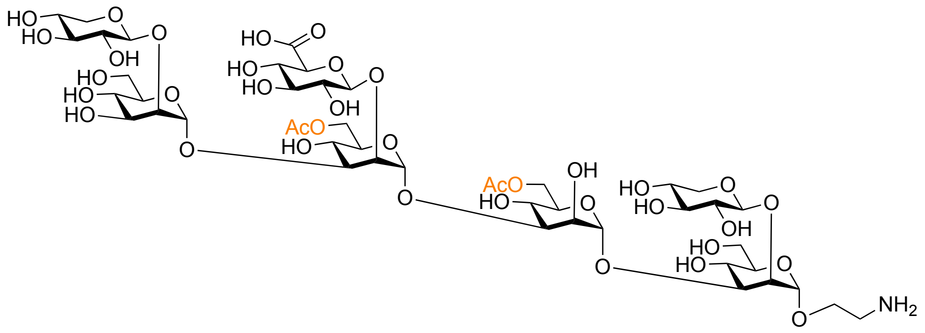

^{1}H NMR (500 MHz, D_2O) δ 5.11 (s, 2H), 4.97 (s, 1H), 4.89 (s, 1H), 4.43 – 4.36 (m, 2H), 4.36 – 4.04 (m, 9H), 4.05 – 3.80 (m, 10H), 3.81 – 3.61 (m, 13H), 3.61 – 3.35 (m, 13H), 3.35 – 3.27 (m, 4H), 3.27 – 3.07 (m, 5H), 3.05 – 2.83 (m, 1H), 2.09 – 2.02 (m, 6H). **^{13}C NMR** (126 MHz, d_2o) δ 100.6, 102.6, 97.9, 102.1, 63.7, 63.1, 103.1, 102.5, 77.6, 63.8, 70.8, 69.5, 77.4, 77.7, 70.9, 65.1, 65.2, 78.8, 73.1, 69.3, 66.4, 60.3, 60.2, 65.9, 73.2, 77.4, 69.2, 61.4, 71.6, 67.5, 75.4, 72.5, 65.1, 20.3. **HRMS** $[M+Na]^+$ *m/z* Calcd for $C_{46}H_{75}NO_{37}Na$, 1256.3916; found, 1256.4020.

2-Aminoethyl β-D-glucopyranosyluronic acid-(1→2)-6-*O*-acetyl-α-D-mannopyranosyl-(1→3)-6-*O*-acetyl-α-D-mannopyranosyl-(1→3)-[β-D-xylo-pyranosyl-(1→2)]-α-D-mannopyranoside-[β-D-glucopyranosyluronic acid-(1→2)]-6-*O*-acetyl-α-D-mannopyranosyl-(1→3)-6-*O*-acetyl-α-D-mannopyranosyl-(1→3)-[β-D-xylopyranosyl-(1→2)]-α-D-mannopyranoside (7)

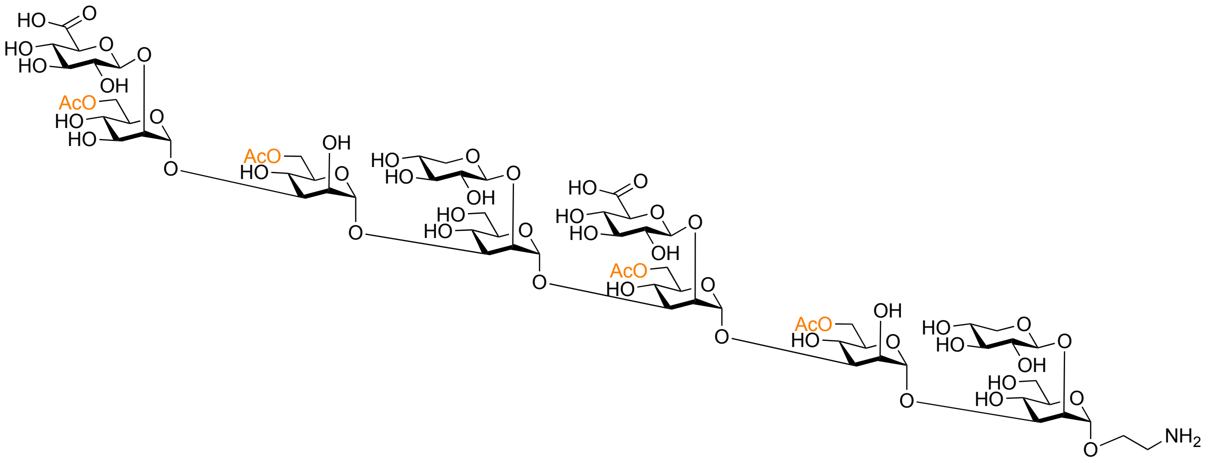

^{1}H NMR (500 MHz, D_2O) δ 5.13 (s, 2H), 5.11 – 5.05 (m, 2H), 4.97 (s, 1H), 4.91 (s, 1H), 4.53 – 4.20 (m, 8H), 4.20 – 3.96 (m, 2H), 3.96 – 3.82 (m, 7H), 3.82 – 3.59 (m, 11H), 3.59 – 3.45 (m, 15H), 3.45 – 3.25 (m, 10H), 3.25 – 2.93 (m, 4H), 2.17 – 1.97 (m, 13H).**^{13}C NMR** (126 MHz, d_2o) δ 100.5, 100.9, 102.3, 102.7, 97.9, 101.6, 63.7, 63.3, 103.0, 103.1, 76.9, 77.7, 69.6, 70.7, 69.5, 70.6, 78.1, 76.4, 77.6, 70.7, 73.1, 63.4, 78.8, 65.1, 69.5, 65.8, 60.2, 66.8, 63.4, 76.0, 67.1, 73.2, 69.2, 77.0, 60.9, 61.4, 71.5, 71.7, 75.2, 75.5, 72.5, 72.6, 39.0, 65.1, 20.4, 20.3. **HRMS** $[M+Na]^+$ *m/z* Calcd for $C_{68}H_{107}NO_{55}Na$, 1840,5504; found, 1840.5587.

2-Aminoethyl β-D-xylopyranosyl-(1→2)-α-D-mannopyranoside-[β-D-glucopyranosyluronic acid-(1→2)]-6-*O*-acetyl-α-D-mannopyranosyl-(1→3)-6-*O*-acetyl-α-D-mannopyranosyl-(1→3)-[β-D-xylopyranosyl-(1→2)]-α-D-mannopyranoside-[β-D-glucopyranosyluronic acid-(1→2)]-6-*O*-acetyl-α-D-mannopyranosyl-(1→3)-6-*O*-acetyl-α-D-mannopyranosyl-(1→3)-[β-D-xylo-pyranosyl-(1→2)]-α-D-mannopyranoside (8)

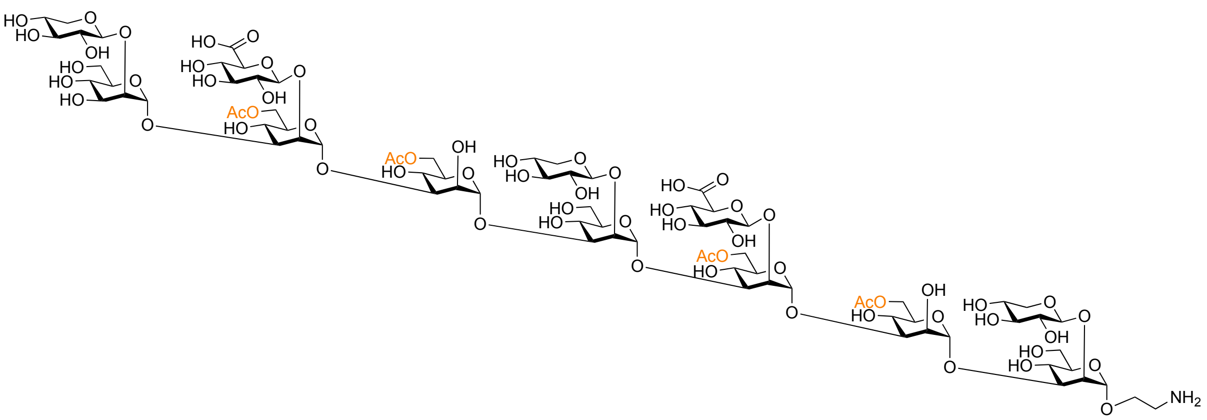

^{1}H NMR (600 MHz, D_2O) δ 5.15 – 4.98 (m, 5H), 4.94 (s, 1H), 4.88 (s, 1H), 4.43 – 4.18 (m, 9H), 4.18 – 4.02 (m, 8H), 4.02 – 3.79 (m, 13H), 3.79 – 3.57 (m, 13H), 3.57 – 3.37 (m, 14H), 3.38 – 3.32 (m, 3H), 3.32 – 3.23 (m, 7H), 3.24 – 3.08 (m, 11H), 2.13 – 1.94 (m, 12H). **^{13}C NMR** (151 MHz, d_2o) δ 100.4, 100.7, 100.6, 102.1, 101.9, 102.6, 97.9, 101.5, 102.3, 63.6, 63.6, 63.0, 63.2, 103.1, 63.1, 102.4, 103.2, 76.8, 63.6, 77.8, 70.8, 78.1, 69.5, 70.7, 69.5, 70.6, 77.3, 76.7, 69.6, 78.0, 76.4, 77.7, 70.8, 75.2, 73.0, 76.1, 76.7, 63.5, 78.7, 65.0, 73.0, 61.2, 69.4, 60.9, 65.7, 66.3, 60.2, 73.3, 60.3, 63.5, 61.0, 73.2, 67.0, 77.3, 76.9, 69.1, 71.5, 67.5, 75.3, 72.3, 75.4, 75.4, 72.3, 72.5, 72.6, 65.1, 39.0, 65.0, 20.3, 20.2. **HRMS** $[M+Na]^+$ *m/z* Calcd for $C_{79}H_{125}NO_{64}Na$, 2134.6455; found, 2134.6421.

2-Aminoethyl 6-*O*-acetyl-α-D-mannopyranosyl-(1→3)-[β-D-xylopyranosyl-(1→2)]-α-D-mannopyranoside-[β-D-glucopyranosyluronic acid-(1→2)]-6-*O*-acetyl-α-D-mannopyranosyl-(1→3)-6-*O*-acetyl-α-D-mannopyranosyl-(1→3)-[β-D-xylopyranosyl-(1→2)]-α-D-mannopyranoside-[β-D-glucopyranosyluronic acid-(1→2)]-6-*O*-acetyl-α-D-mannopyranosyl-(1→3)-6-*O*-acetyl-α-D-mannopyranosyl-(1→3)-[β-D-xylopyranosyl-(1→2)]-α-D-mannopyranoside (9)

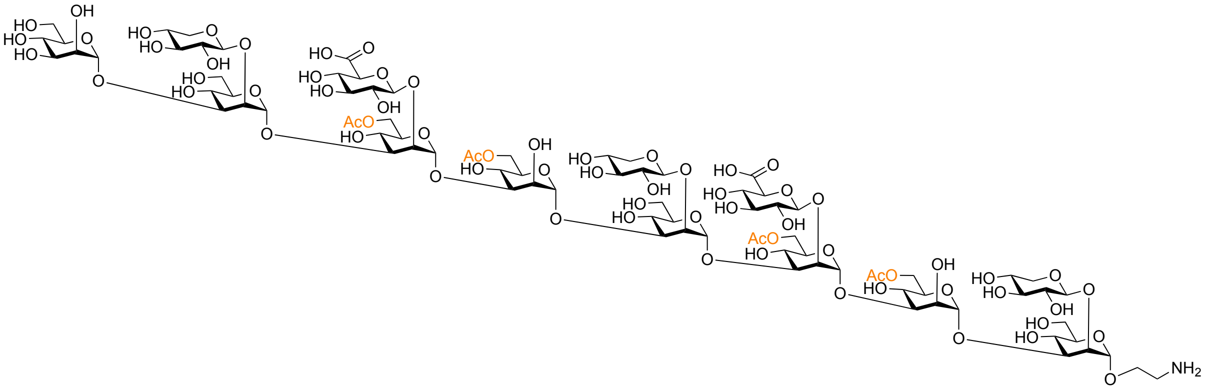

^{1}H NMR (500 MHz, D_2O) δ 5.20 – 4.99 (m, 5H), 4.95 (s, 1H), 4.89 (s, 1H), 4.78 (s, 1H), 4.46 – 4.32 (m, 4H), 4.31 – 4.04 (m, 11H), 4.04 – 3.81 (m, 15H), 3.81 – 3.62 (m, 16H), 3.62 – 3.43 (m, 12H), 3.41 – 3.24 (m, 7H), 3.24 – 3.01 (m, 16H), 2.69 (t, J = 10.6 Hz, 1H), 2.15 – 1.99 (m, 16H). **^{13}C NMR** (126 MHz, d_2o) δ 100.4, 100.4, 103.1, 102.1, 102.7, 97.9, 74.3, 74.3, 101.7, 63.2, 103.2, 77.7, 103.2, 77.0, 63.5, 63.0, 70.8, 69.5, 70.5, 78.1, 69.8, 77.7, 70.8, 73.2, 63.5, 78.3, 76.7, 65.2, 78.6, 73.3, 102.7, 65.9, 60.2, 66.4, 63.7, 73.2, 77.2, 66.9, 69.2, 71.5, 72.3, 75.5, 72.5, 39.1, 72.5, 73.2, 75.2, 65.0, 64.9, 20.4, 20.2. **HRMS** $[M+H+Na]^{+2}$ *m/z* Calcd for $C_{85}H_{136}NO_{69}Na$, 1148.8526; found, 1148.8520.

2-Aminoethyl β-D-glucopyranosyluronic acid-(1→2)-6-*O*-acetyl-α-D-mannopyranosyl-(1→3)-6-*O*-acetyl-α-D-mannopyranosyl-(1→3)-[β-D-xylo-pyranosyl-(1→2)]-α-D-mannopyranoside-[β-D-glucopyranosyluronic acid-(1→2)]-6-*O*-acetyl-α-D-mannopyranosyl-(1→3)-6-*O*-acetyl-α-D-mannopyranosyl-(1→3)-[β-D-xylopyranosyl-(1→2)]-α-D-mannopyranoside-[β-D-glucopyranosyluronic acid-(1→2)]-6-*O*-acetyl-α-D-mannopyranosyl-(1→3)-6-*O*-acetyl-α-D-mannopyranosyl-(1→3)-[β-D-xylopyranosyl-(1→2)]-α-D-mannopyranoside (10)

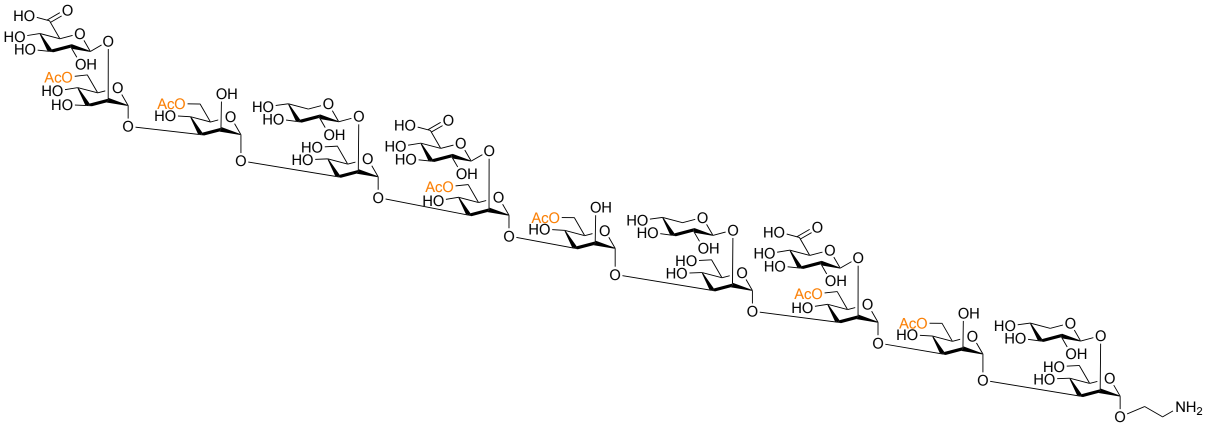

^{1}H NMR (600 MHz, D_2O) δ 5.12 – 5.01 (m, 7H), 4.94 (s, 1H), 4.88 (s, 1H), 4.41 – 4.33 (m, 5H), 4.32 – 4.27 (m, 4H), 4.27 – 4.20 (m, 16H), 4.20 – 4.15 (m, 2H), 4.07 – 3.96 (m, 3H), 3.97 – 3.79 (m, 23H), 3.80 – 3.56 (m, 19H), 3.57 – 3.42 (m, 11H), 3.42 – 3.33 (m, 4H), 3.31 – 3.23 (m, 9H), 3.24 – 3.02 (m, 13H), 2.10 – 1.98 (m, 20H). **^{13}C NMR** (151 MHz, d_2o) δ 100.3, 100.8, 102.2, 102.5, 97.9, 101.7, 63.3, 103.2, 103.2, 76.9, 77.7, 69.4, 70.6, 70.4, 77.9, 76.4, 61.1, 77.6, 70.8, 63.3, 73.1, 76.2, 64.9, 78.7, 78.6, 63.9, 69.4, 61.1, 65.8, 60.1, 65.9, 63.8, 68.3, 75.7, 63.9, 73.1, 66.9, 76.9, 69.1, 71.4, 71.6, 75.1, 75.4, 72.3, 72.6, 64.9, 39.0, 20.2. **HRMS** $[M+H]^+$ *m/z* Calcd for $C_{101}H_{158}NO_{82}$, 2696.82219; found, 2696.8220.

2-Aminoethyl β-D-xylopyranosyl-(1→2)-6-*O*-acetyl-α-D-mannopyranosyl-(1→3)-[β-D-glucopyranosyluronic acid-(1→2)]-6-*O*-acetyl-α-D-mannopyranosyl-(1→3)-[β-D-xylopyranosyl-(1→2)]-α-D-mannopyranosyl-(1→3)-[β-D-xylopyranosyl-(1→2)]-6-*O*-acetyl-α-D-mannopyranoside (14)

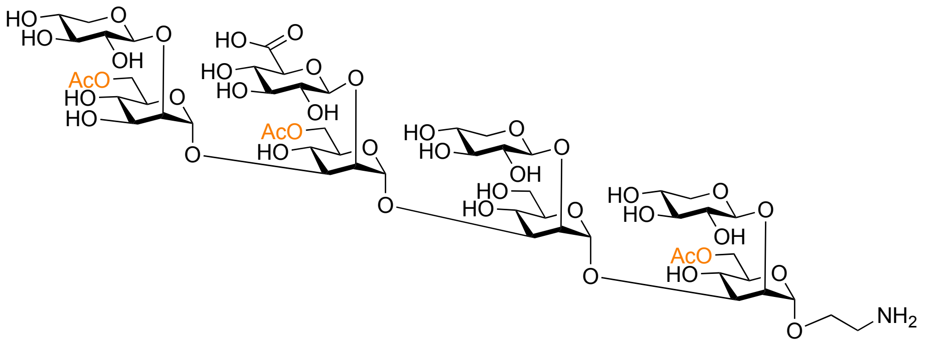

^{1}H NMR (500 MHz, D_2O) δ 5.13 (s, 1H), 5.09 (s, 1H), 4.85 (s, 1H), 4.35 – 4.14 (m, 4H), 4.14 – 3.89 (m, 1H), 3.89 – 3.62 (m, 2H), 3.59 – 3.41 (m, 3H), 3.39 – 3.25 (m, 3H), 3.25 – 3.04 (m, 1H), 2.13 – 1.98 (m, 8H). **^{13}C NMR** (126 MHz, d_2o) δ 99.9, 101.2, 98.4, 102.5, 103.4, 63.2, 103.2, 102.9, 77.6, 70.3, 77.8, 74.1, 75.1, 75.3, 76.6, 65.0, 69.3, 73.5, 70.7, 66.5, 60.4, 60.4, 64.0, 77.5, 67.7, 69.2, 71.5, 69.2, 75.3, 72.3, 72.7, 65.0, 39.0, 20.4, 20.4, 20.0. **HRMS** $[M+H]^+$ *m/z* Calcd for $C_{53}H_{86}NO_{42}$, 1408,4619; found, 1408.4622.

2-Aminoethyl β-D-glucopyranosyluronic acid-(1→2)-6-*O*-acetyl-α-D-mannopyranosyl-(1→3)-[β-D-xylopyranosyl-(1→2)]-α-D-mannopyranosyl-(1→3)-[(β-D-xylopyranosyl)-(1→2)]-6-*O*-acetyl-α-D-mannopyranosyl-(1→3)-[β-D-glucopyranosyluronic acid-(1→2)]-6-*O*-acetyl-α-D-mannopyranosyl-(1→3)-[β-D-xylopyranosyl-(1→2)]-α-D-mannopyranosyl-(1→3)-[β-D-xylopyranosyl-(1→2)]-6-*O*-acetyl-α-D-mannopyranoside (16)

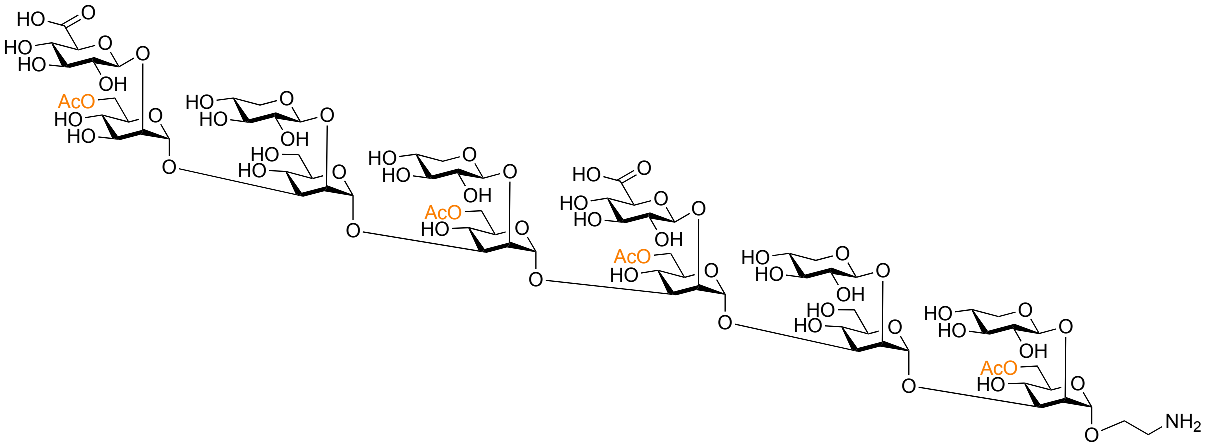

^{1}H NMR (600 MHz, D_2O) δ 5.12 – 5.01 (m, 4H), 4.94 (s, 1H), 4.88 (s, 1H), 4.41 – 4.33 (m, 5H), 4.32 – 4.27 (m, 4H), 4.27 – 4.20 (m, 16H), 4.20 – 4.15 (m, 2H), 4.07 – 3.96 (m, 3H), 3.97 – 3.79 (m, 23H), 3.80 – 3.56 (m, 19H), 3.57 – 3.42 (m, 11H), 3.42 – 3.33 (m, 4H), 3.31 – 3.23 (m, 9H), 3.24 – 3.02 (m, 13H), 2.10 – 1.98 (m, 12H). **^{13}C NMR** (151 MHz, d_2o) δ 100.3, 100.8, 102.2, 102.5, 97.9, 101.7, 63.3, 103.2, 103.2, 76.9, 77.7, 69.4, 70.6, 70.4, 77.9, 76.4, 61.1, 77.6, 70.8, 63.3, 73.1, 76.2, 64.9, 78.7, 78.6, 63.9, 69.4, 61.1, 65.8, 60.1, 65.9, 63.8, 68.3, 75.7, 63.9, 73.1, 66.9, 76.9, 69.1, 71.4, 71.6, 75.1, 75.4, 72.3, 72.6, 64.9, 39.0, 20.2. **HRMS** $[M+H]^+$ *m/z* Calcd for $C_{78}H_{124}NO_{63}$, 2082.6525; found, 2082.6534.

2-Aminoethyl β-D-xylopyranosyl-(1→2)-6-*O*-acetyl-α-D-mannopyranosyl-(1→3)-[β-D-glucopyranosyluronic acid-(1→2)]-6-*O*-acetyl-α-D-mannopyranosyl-(1→3)-[β-D-xylopyranosyl-(1→2)]-α-D-mannopyranosyl-(1→3)-[(β-D-xylopyranosyl)-(1→2)]-6-*O*-acetyl-α-D-mannopyranosyl-(1→3)-[β-D-glucopyranosyluronic acid-(1→2)]-6-*O*-acetyl-α-D-mannopyranosyl-(1→3)-[β-D-xylopyranosyl-(1→2)]-α-D-mannopyranosyl-(1→3)-[β-D-xylopyranosyl-(1→2)]-6-*O*-acetyl-α-D-mannopyranoside (17)

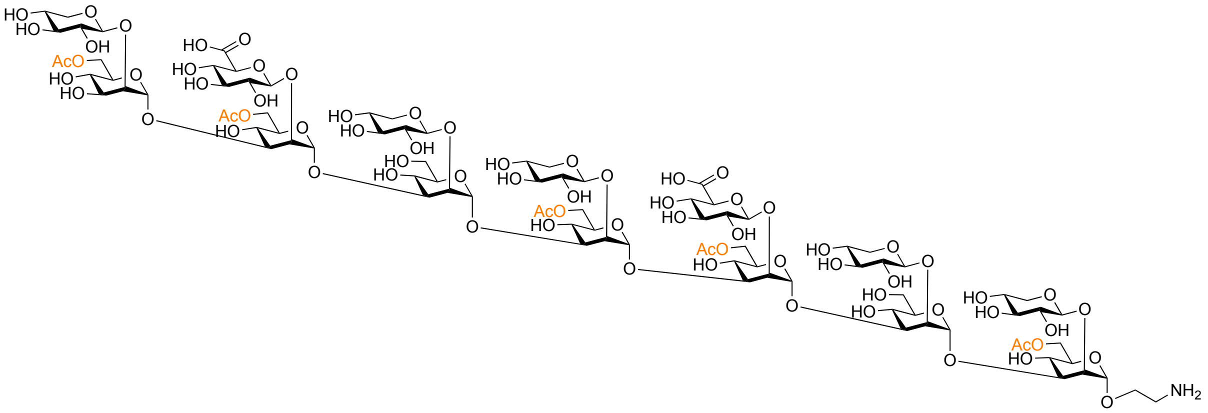

^{1}H NMR (600 MHz, D_2O) δ 5.18 (s, 1H), 5.11 (s, 3H), 5.08 (s, 1H), 4.99 (s, 1H), 4.83 (s, 1H), 4.38 – 4.15 (m, 7H), 4.14 – 3.90 (m, 8H), 3.90 – 3.55 (m, 16H), 3.55 – 3.41 (m, 11H), 3.41 – 3.23 (m, 7H), 3.23 – 3.00 (m, 11H), 2.12 – 1.97 (m, 15H). **^{13}C NMR** (151 MHz, d_2o) δ 99.9, 100.1, 101.2, 101.9, 98.4, 102.4, 103.4, 63.2, 103.0, 63.0, 78.5, 77.8, 78.0, 70.5, 78.0, 75.3, 76.6, 76.8, 78.0, 77.8, 63.9, 65.0, 69.3, 73.3, 60.5, 70.7, 60.4, 66.5, 60.5, 67.3, 63.9, 77.3, 68.7, 67.7, 69.2, 71.5, 75.3, 72.2, 72.7, 72.8, 65.0, 39.0, 38.3, 20.4, 20.4, 20.2. **HRMS** $[M+H+Na]^{+2}$ *m/z* Calcd for $C_{91}H_{144}NO_{73}Na$, 1220.8737; found, 1220.8735.

2-Aminoethyl β-D-xylopyranosyl-(1→2)-α-D-mannopyranosyl-(1→3)-[β-D-xylopyranosyl-(1→2)]-6-*O*-acetyl-α-D-mannopyranosyl-(1→3)-[β-D-glucopyranosyluronic acid-(1→2)]-6-*O*-acetyl-α-D-mannopyranosyl-(1→3)-[β-D-xylopyranosyl-(1→2)]-α-D-mannopyranosyl-(1→3)-[(β-D-xylopyranosyl)-(1→2)]-6-*O*-acetyl-α-D-mannopyranosyl-(1→3)-[β-D-glucopyranosyluronic acid-(1→2)]-6-*O*-acetyl-α-D-mannopyranosyl-(1→3)-[β-D-xylopyranosyl-(1→2)]-α-D-mannopyranosyl-(1→3)-[β-D-xylopyranosyl-(1→2)]-6-*O*-acetyl-α-D-mannopyranoside (18)

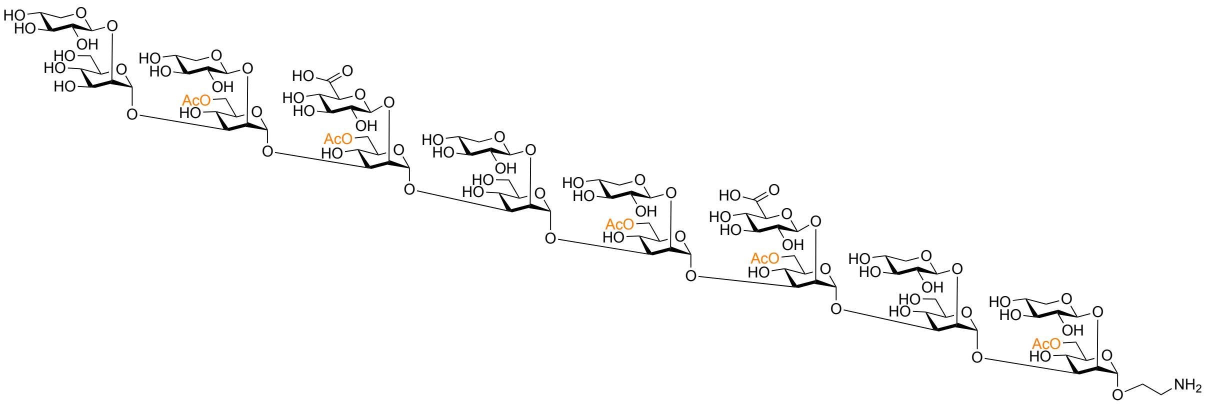

^{1}H NMR (600 MHz, D_2O) δ 5.18 (s, 1H), 5.12 (s, 1H), 5.02 (s, 1H), 4.99 (s, 1H), 4.83 (s, 1H), 4.41 – 4.18 (m, 6H), 4.18 – 3.92 (m, 7H), 3.92 – 3.78 (m, 2H), 3.78 – 3.59 (m, 3H), 3.59 – 3.44 (m, 4H), 3.41 – 3.24 (m, 4H), 3.24 – 3.00 (m, 7H), 2.14 – 1.97 (m, 6H). **^{13}C NMR** (151 MHz, d_2o) δ 100.1, 100.1, 102.2, 102.5, 63.2, 102.9, 103.2, 63.0, 63.2, 63.4, 103.7, 103.5, 78.6, 77.8, 70.5, 70.3, 75.3, 75.2, 77.5, 64.9, 65.2, 69.8, 61.0, 66.4, 66.7, 60.7, 77.3, 69.0, 75.5, 76.1, 75.3, 74.5, 75.2, 74.2, 72.5, 75.3, 75.5, 72.7, 65.2, 65.0, 39.3, 20.5, 20.4, 20.0. **HRMS** $[M+Na]^+$ *m/z* Calcd for $C_{102}H_{161}NO_{82}Na$, 2734.8351; found, 2734.8356.

2-Aminoethyl β-D-glucopyranosyluronic acid-(1→2)-6-*O*-acetyl-α-D-mannopyranosyl-(1→3)-[β-D-xylopyranosyl-(1→2)]-α-D-mannopyranosyl-(1→3)-[β-D-xylopyranosyl-(1→2)]-6-*O*-acetyl-α-D-mannopyranosyl-(1→3)-[β-D-glucopyranosyluronic acid-(1→2)]-6-*O*-acetyl-α-D-mannopyranosyl-(1→3)-[β-D-xylopyranosyl-(1→2)]-α-D-mannopyranosyl-(1→3)-[(β-D-xylopyranosyl)-(1→2)]-6-*O*-acetyl-α-D-mannopyranosyl-(1→3)-[β-D-glucopyranosyluronic acid-(1→2)]-6-*O*-acetyl-α-D-mannopyranosyl-(1→3)-[β-D-xylopyranosyl-(1→2)]-α-D-mannopyranosyl-(1→3)-[β-D-xylopyranosyl-(1→2)]-6-*O*-acetyl-α-D-mannopyranoside (26)

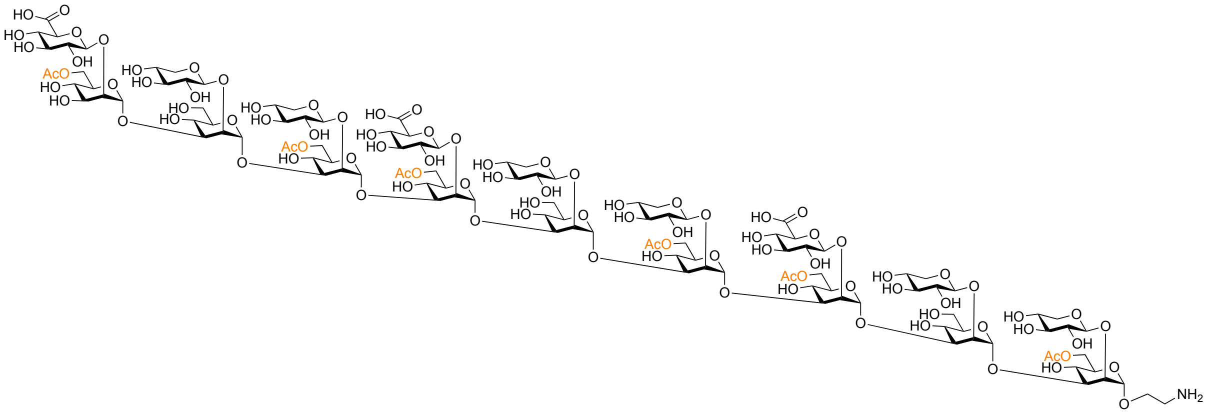

^{1}H NMR (500 MHz, D_2O) δ 5.21 (s, 1H), 5.15 (s, 2H), 5.02 (s, 2H), 4.86 (s, 1H), 4.42 – 4.19 (m, 17H), 4.23 – 4.00 (m, 14H), 4.00 – 3.83 (m, 9H), 3.83 – 3.73 (m, 6H), 3.73 – 3.58 (m, 9H), 3.51 (d, *J* = 20.9 Hz, 8H), 3.44 – 3.25 (m, 8H), 3.25 – 3.11 (m, 10H), 2.13 – 1.99 (m, 14H). **^{13}C NMR** (126 MHz, d_2o) δ 99.8, 100.0, 100.7, 101.9, 98.3, 103.3, 102.1, 63.4, 103.6, 78.3, 77.7, 74.5, 65.0, 73.5, 66.4, 60.6, 67.4, 75.9, 77.2, 69.2, 71.5, 71.6, 72.2, 75.4, 72.3, 72.7, 65.1, 39.0, 20.4, 20.4, 20.1. **HRMS** $[M+Na]^+$ *m/z* Calcd for $C_{116}H_{181}NO_{94}Na$, 3114.9306; found, 3114.9319.

NMR Spectra

Compound 5

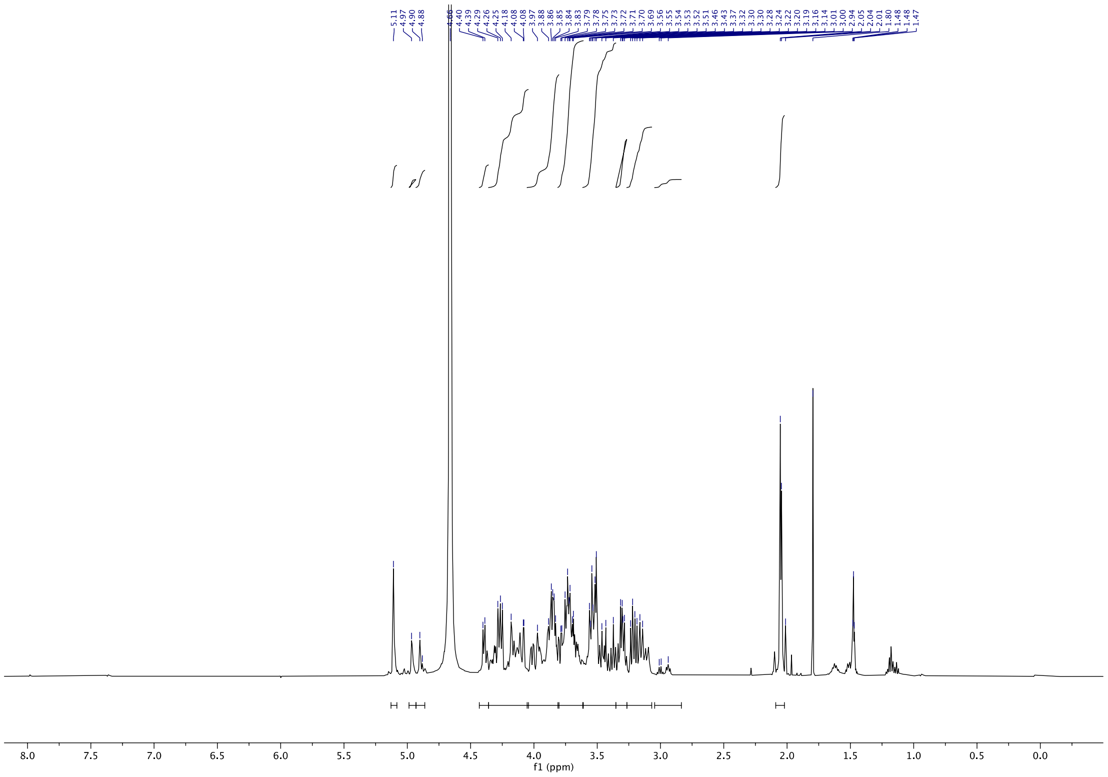

^{1}H NMR

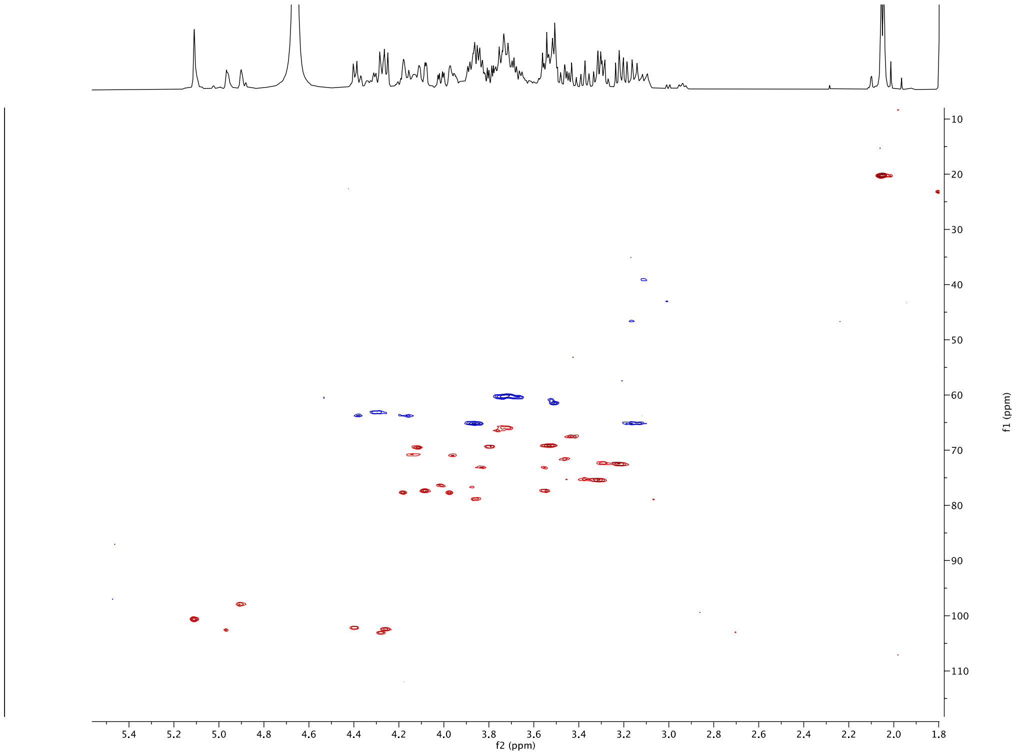

^{1}H-^{13}C HSQC NMR

Compound 7

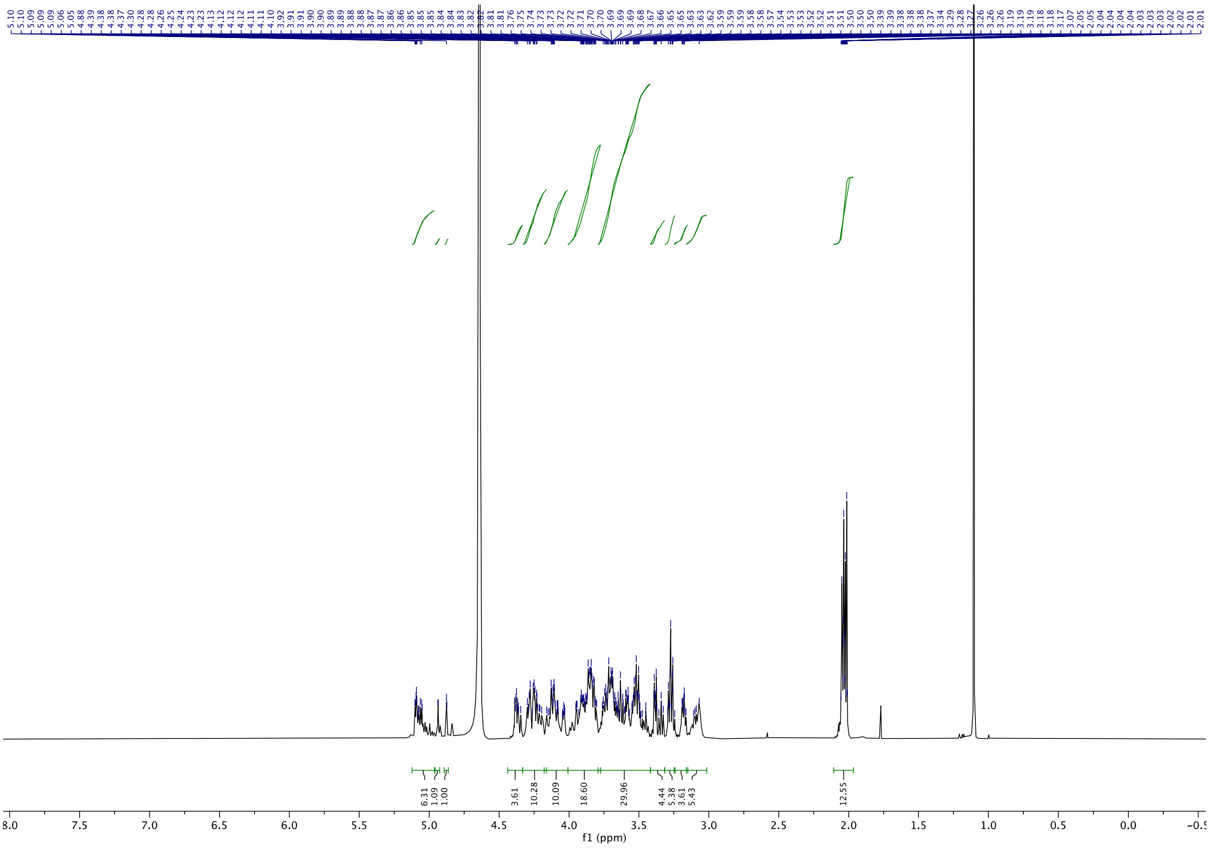

^{1}H NMR

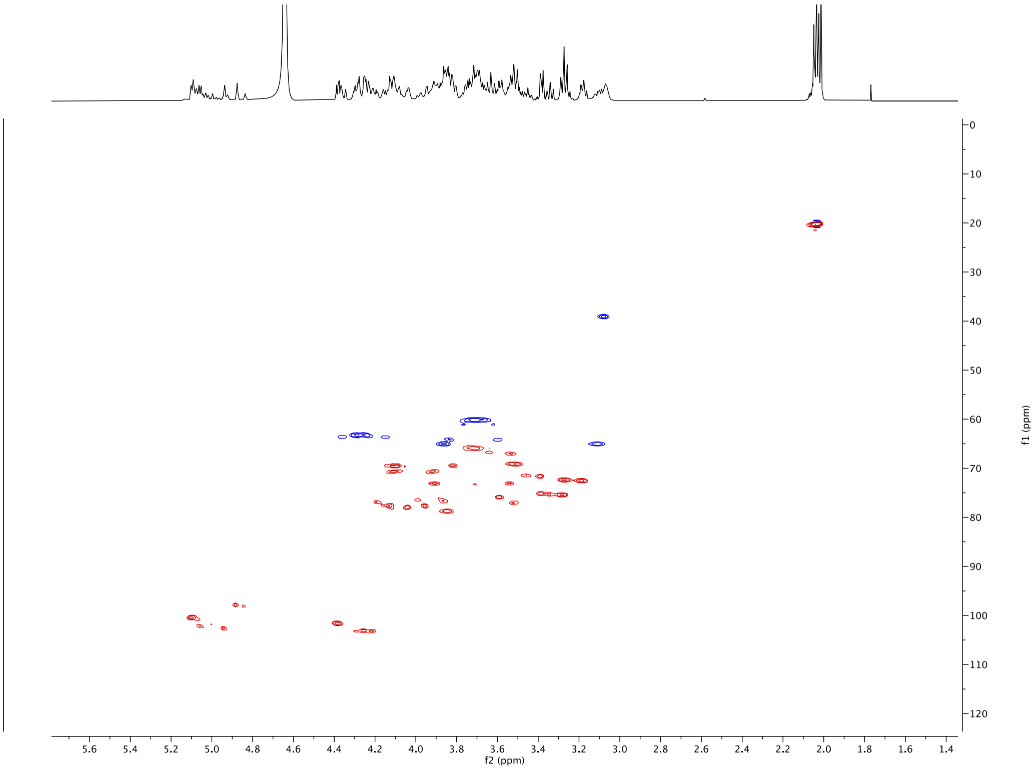

^{1}H-^{13}C HSQC NMR

Compound 8

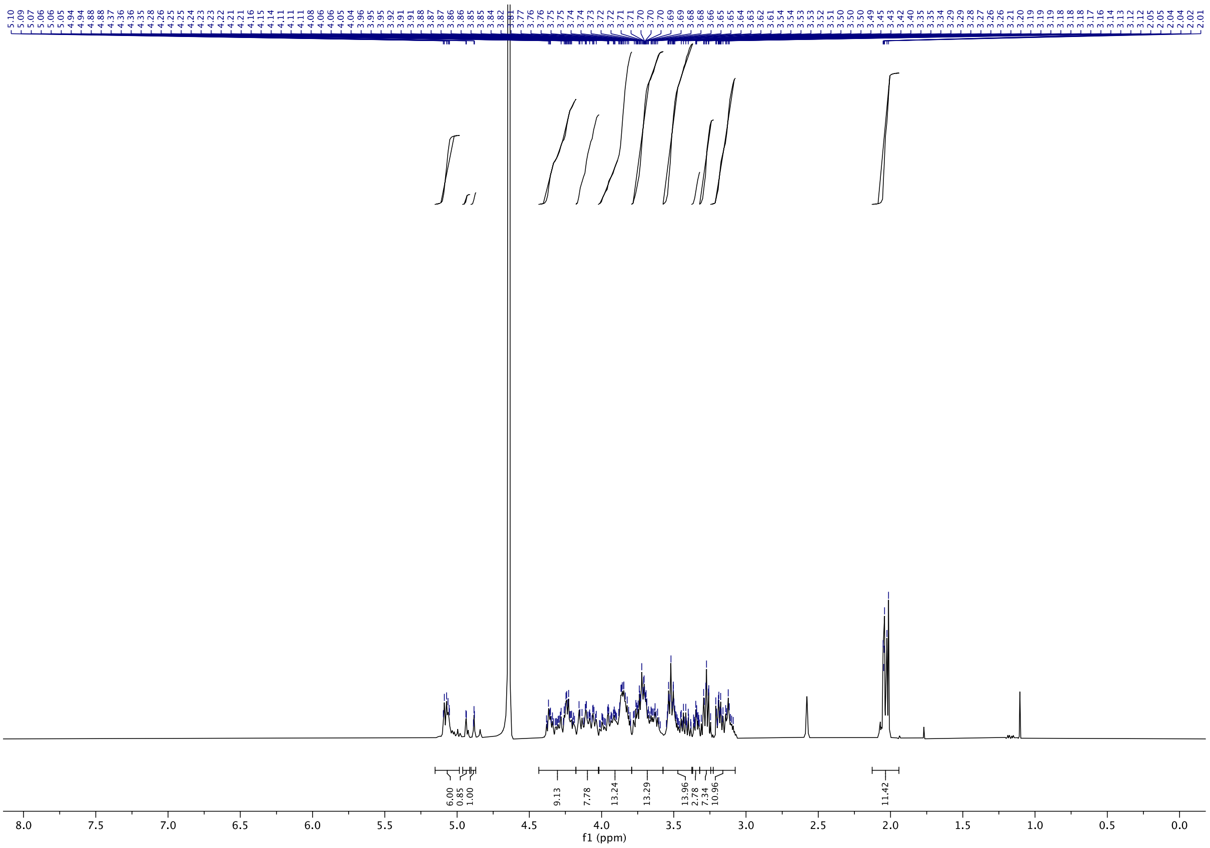

^{1}H NMR

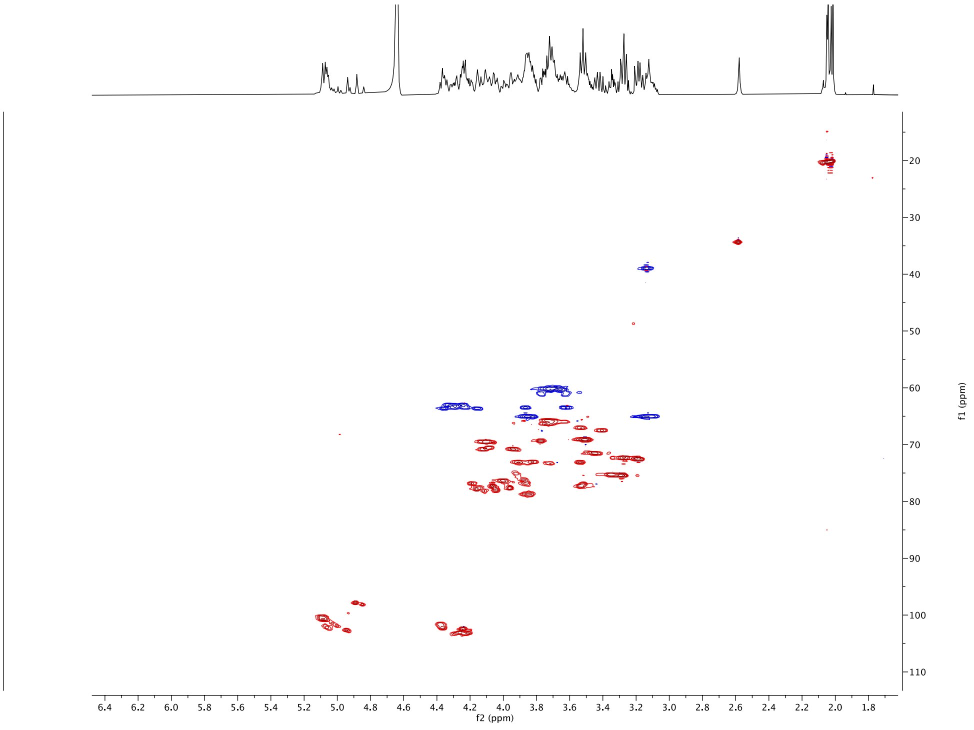

^{1}H-^{13}C HSQC NMR

Compound 9

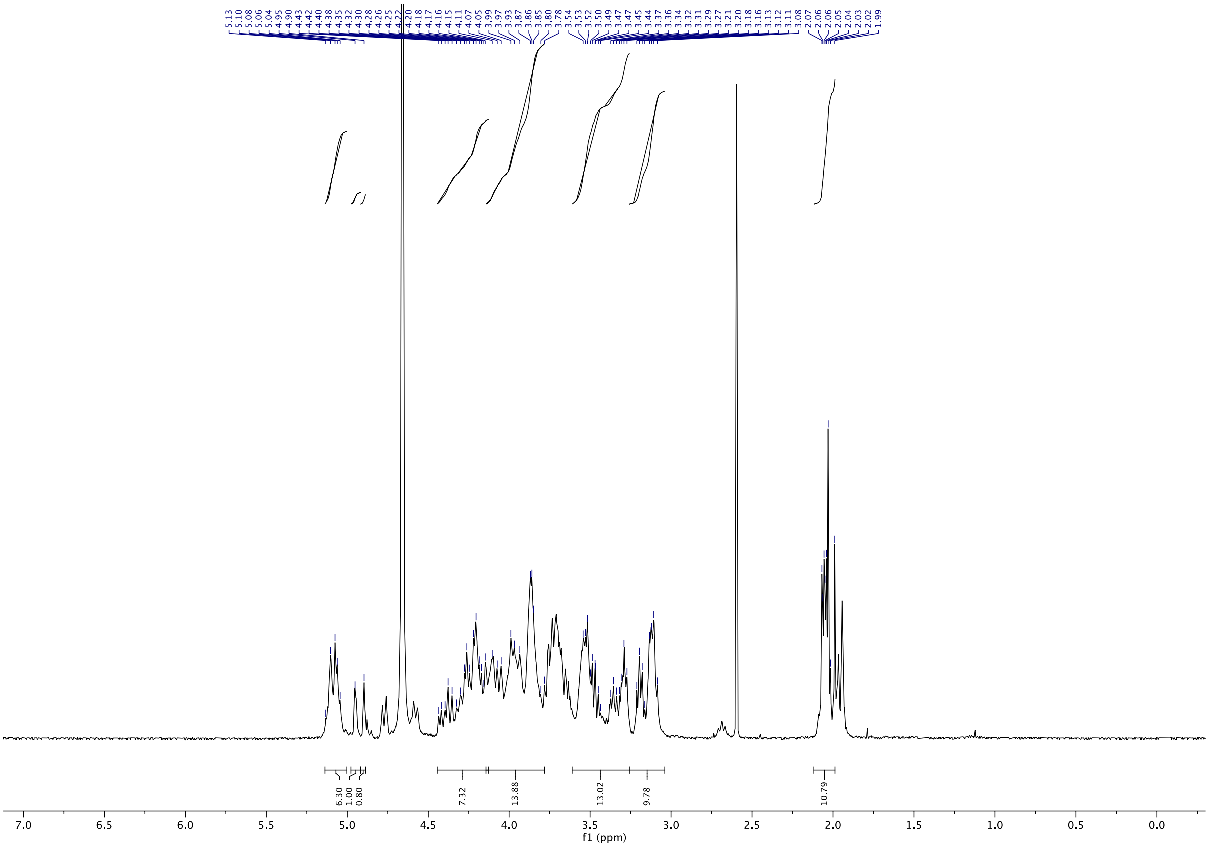

^{1}H NMR

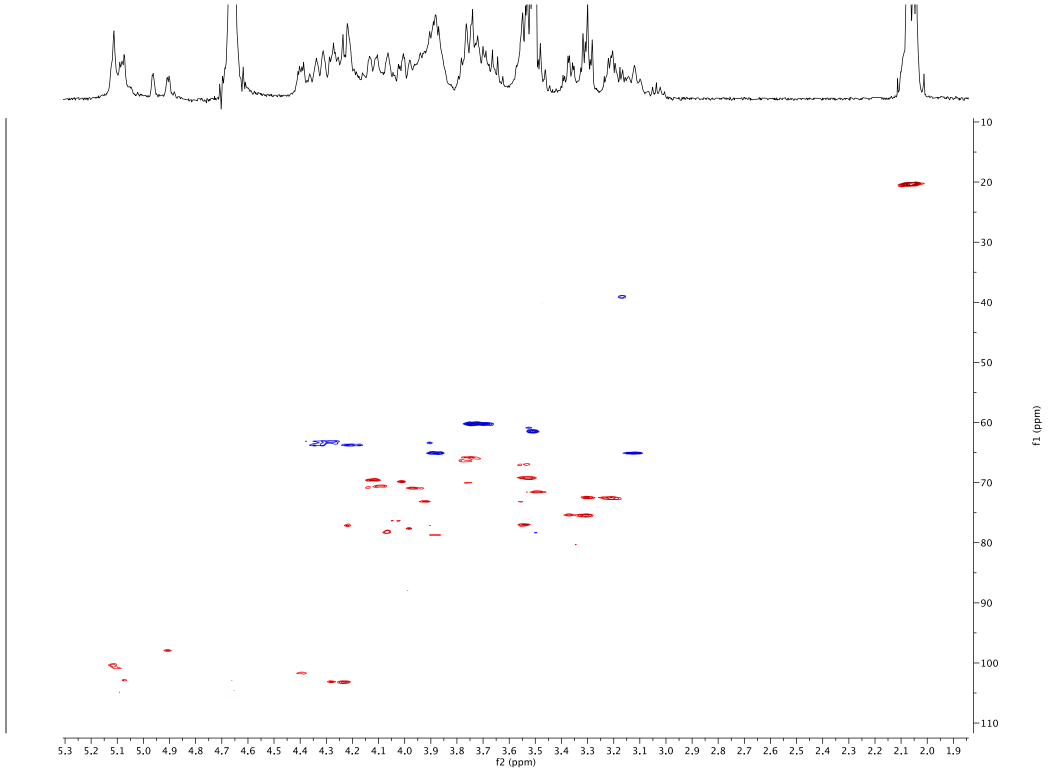

^{1}H-^{13}C HSQC NMR

Compound 10

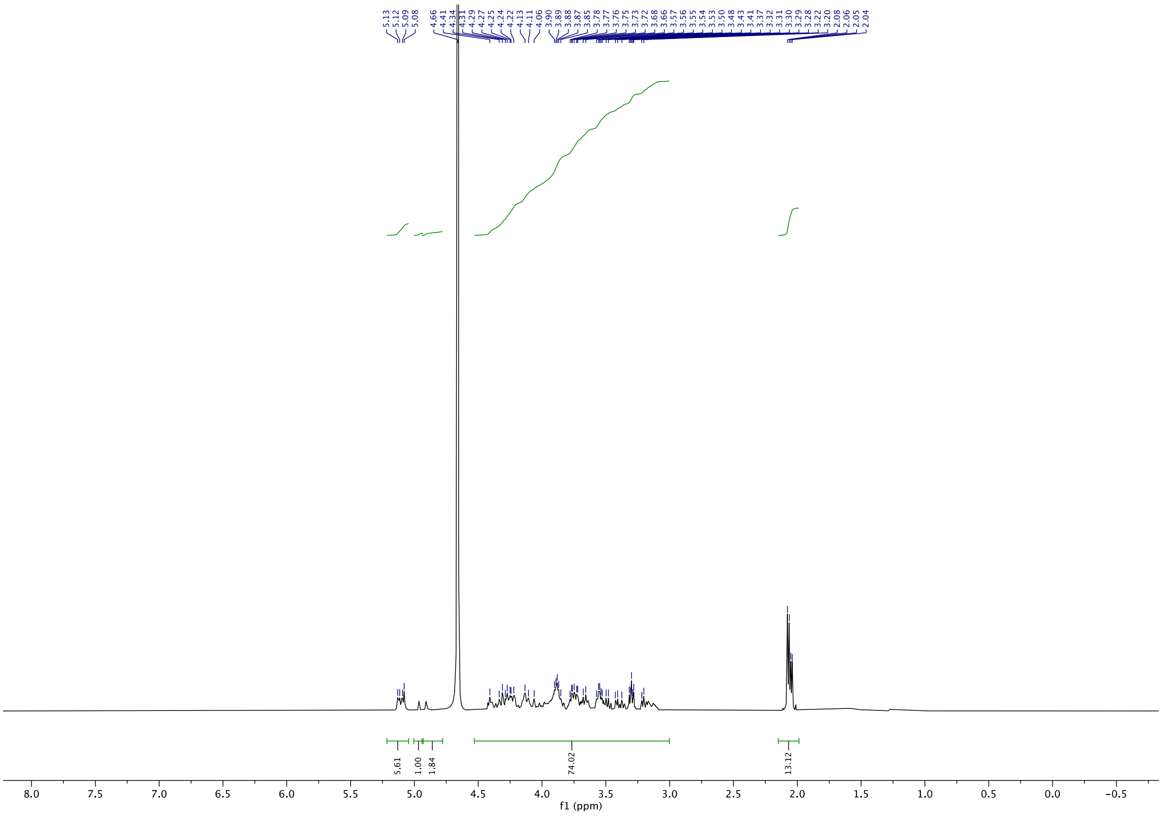

^{1}H NMR

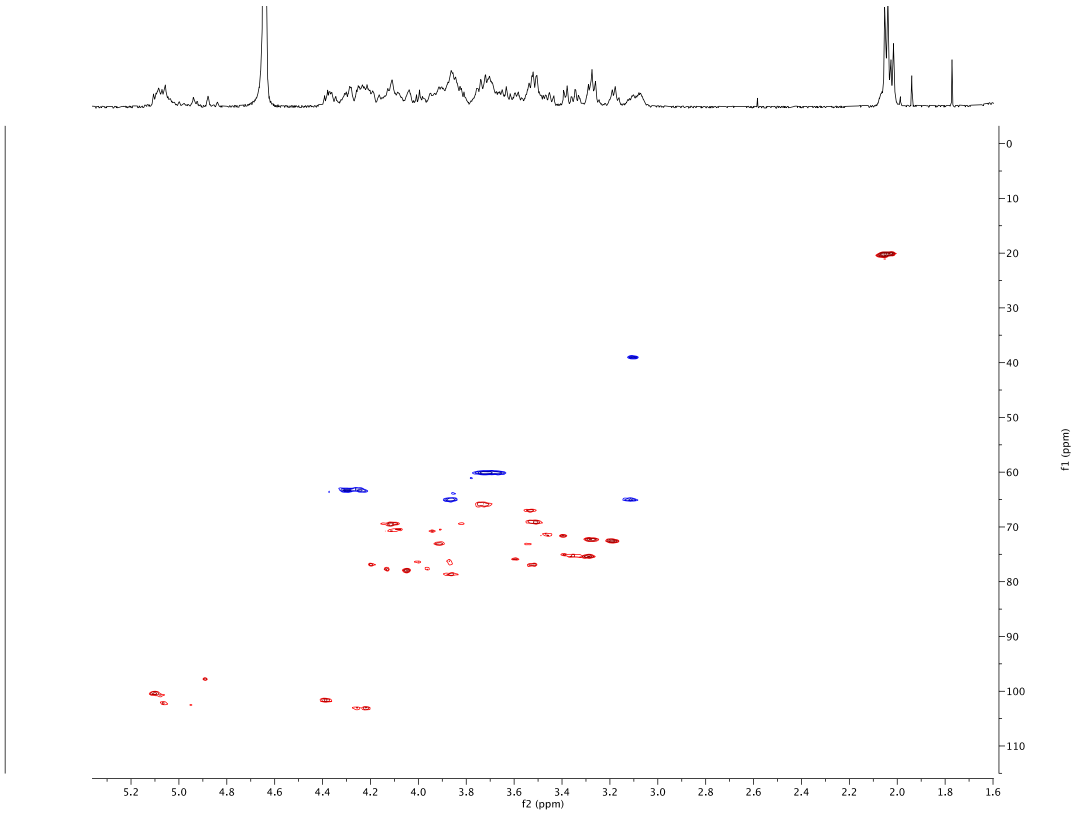

^{1}H-^{13}C HSQC NMR

Compound 14

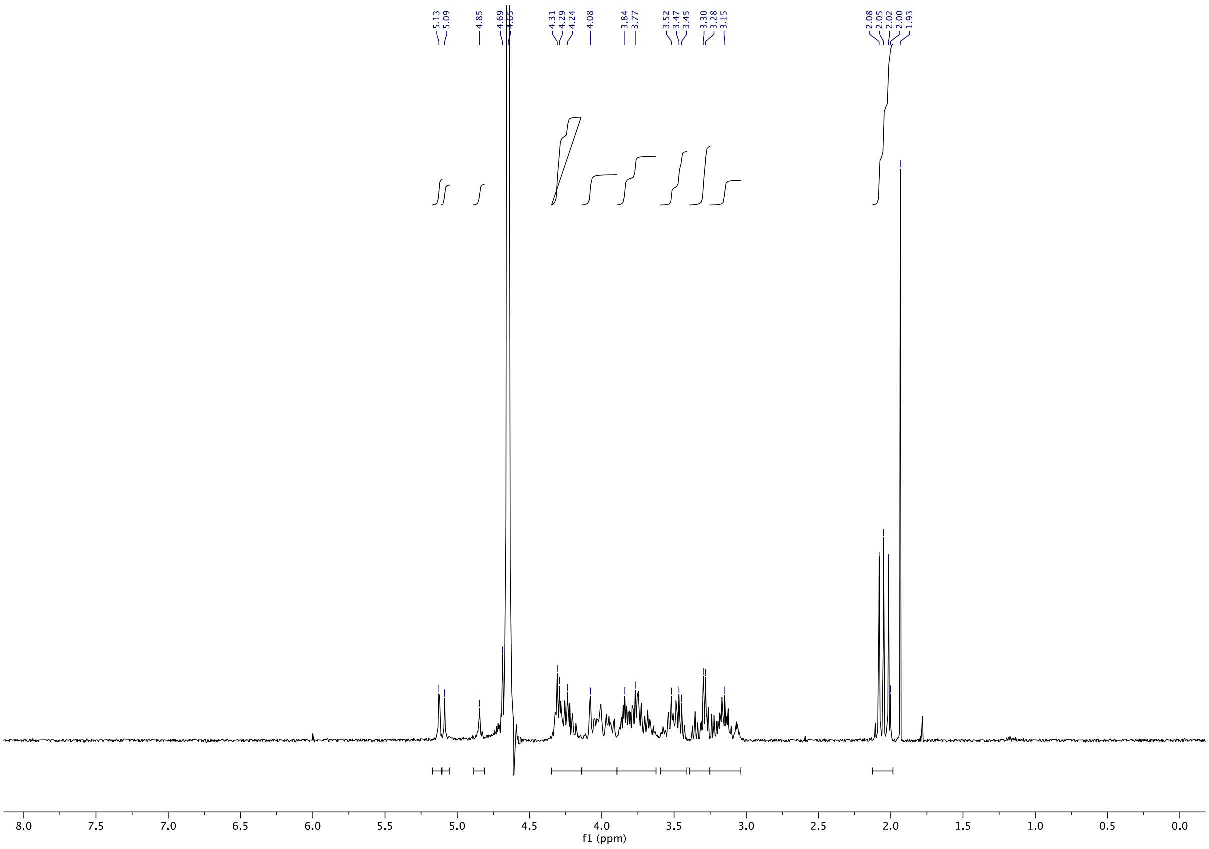

^{1}H NMR

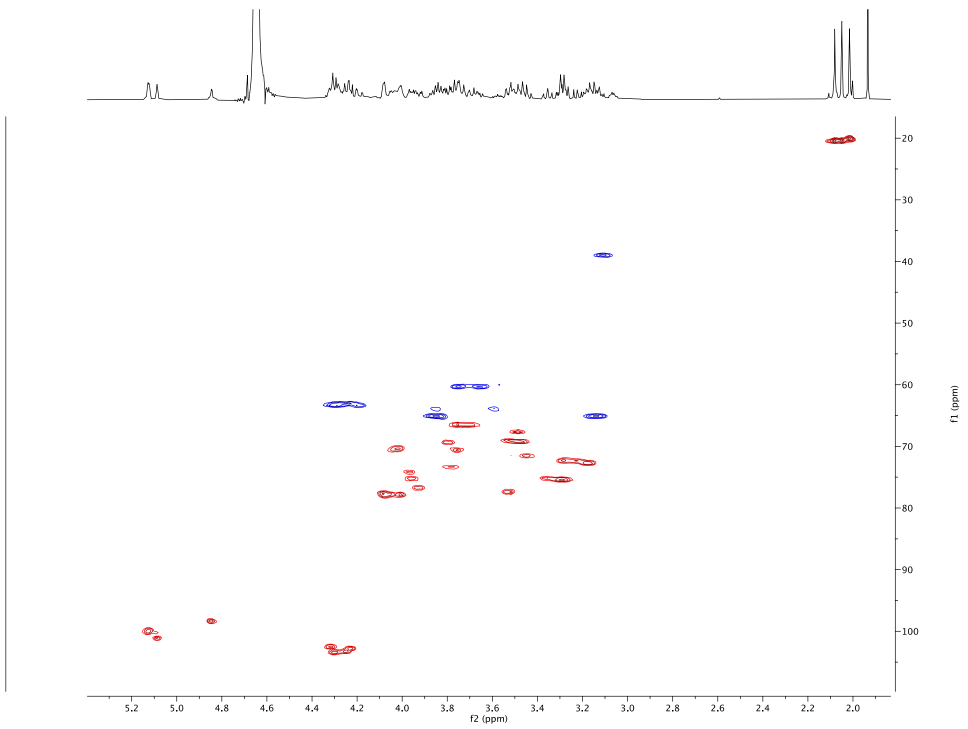

^{1}H-^{13}C HSQC NMR

Compound 16

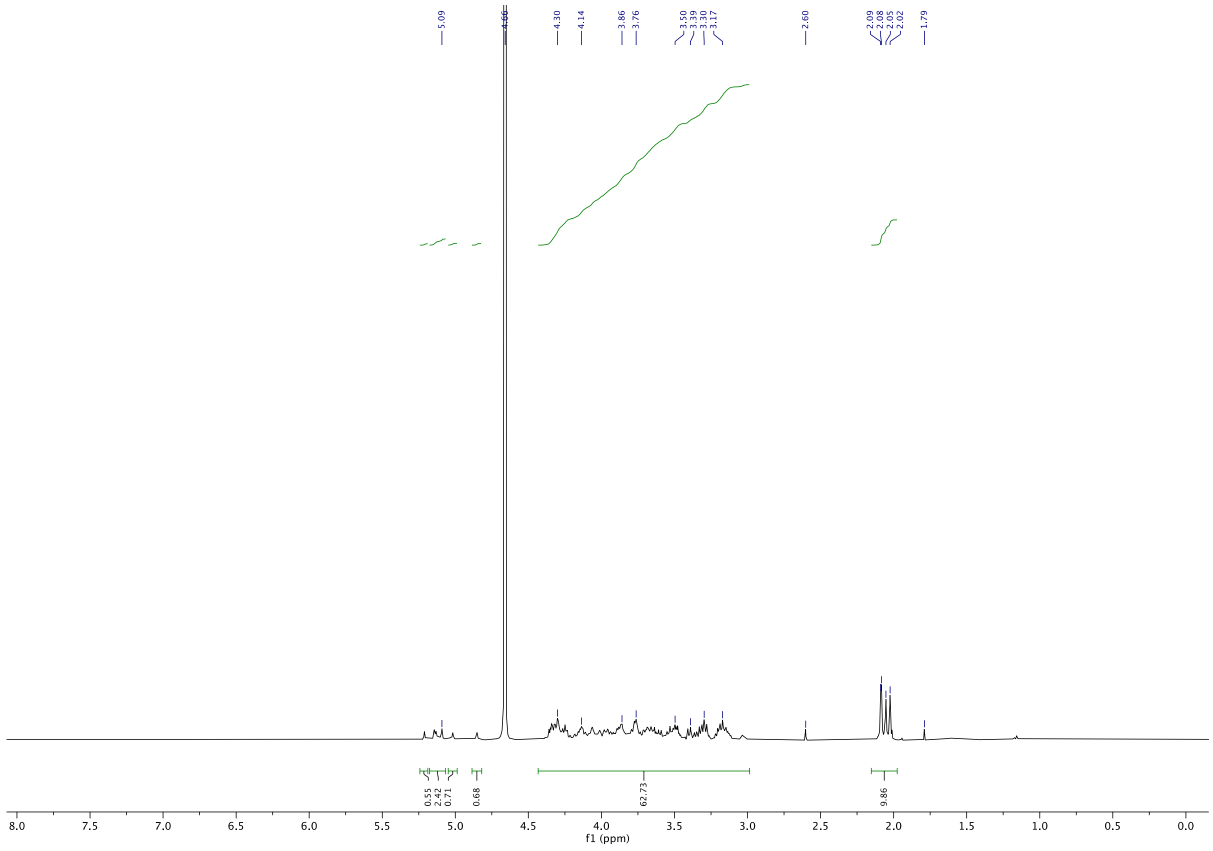

^{1}H NMR

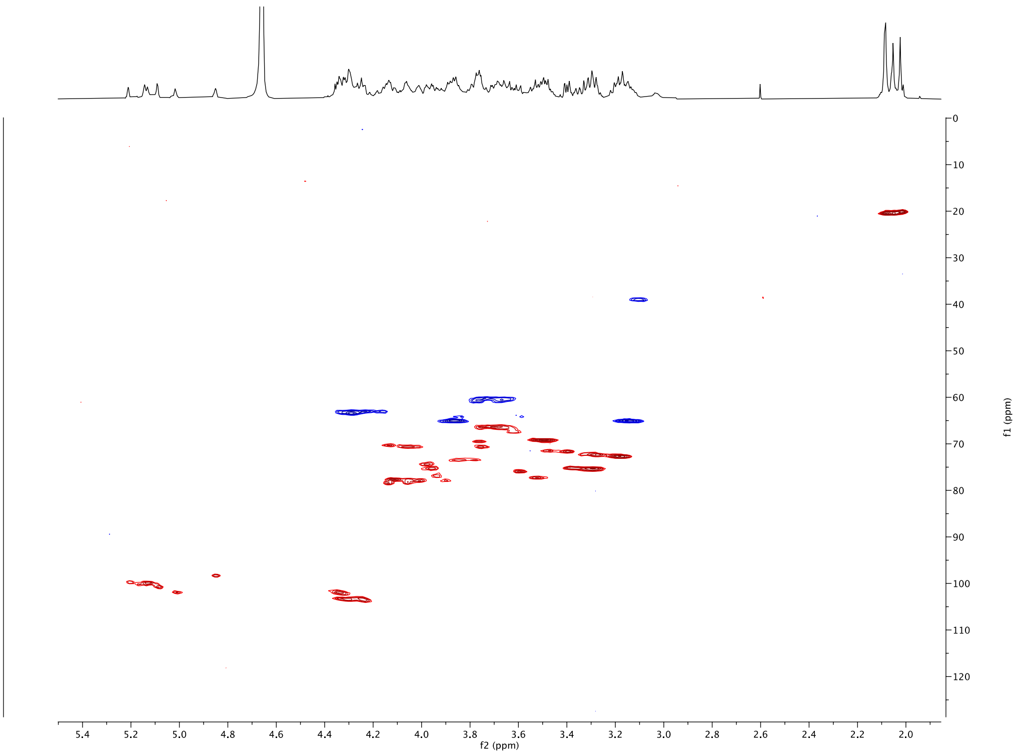

^1H-^{13}C HSQC NMR

Compound 17

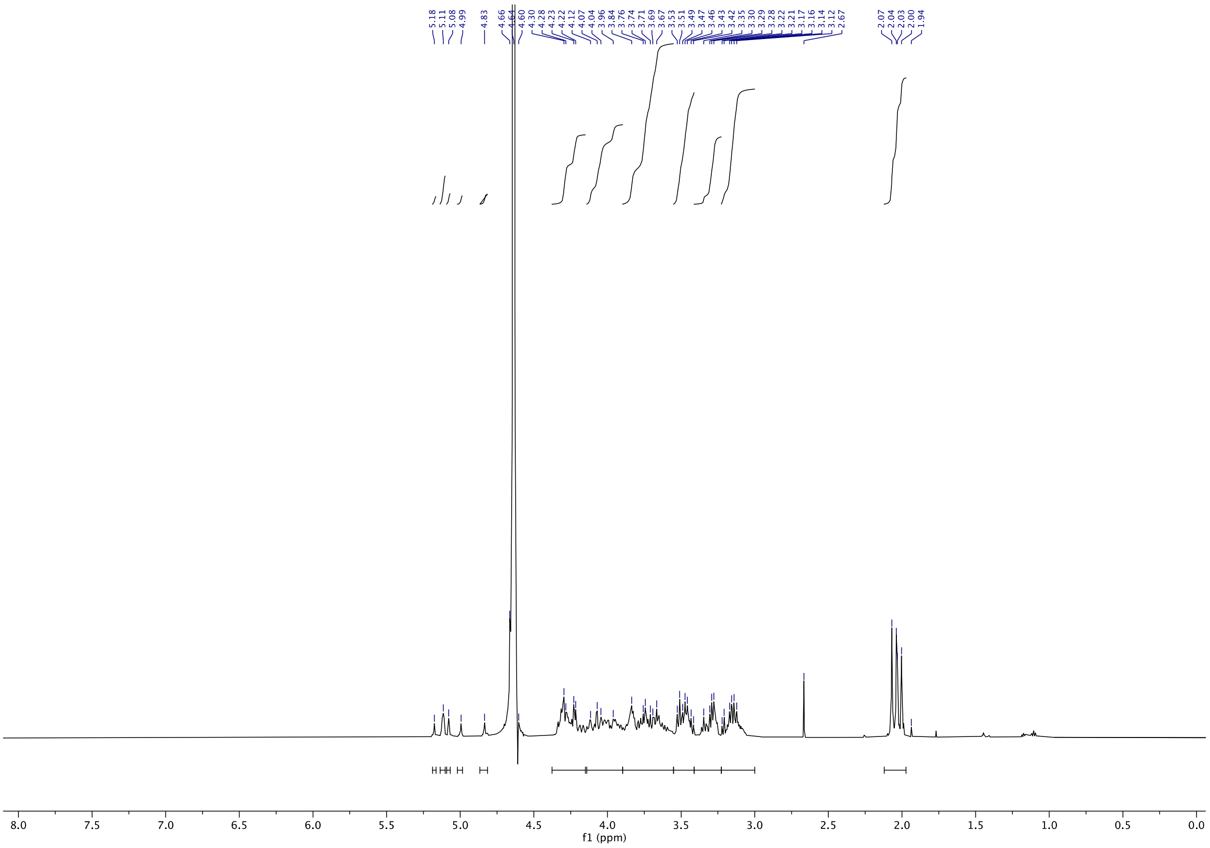

^1H NMR

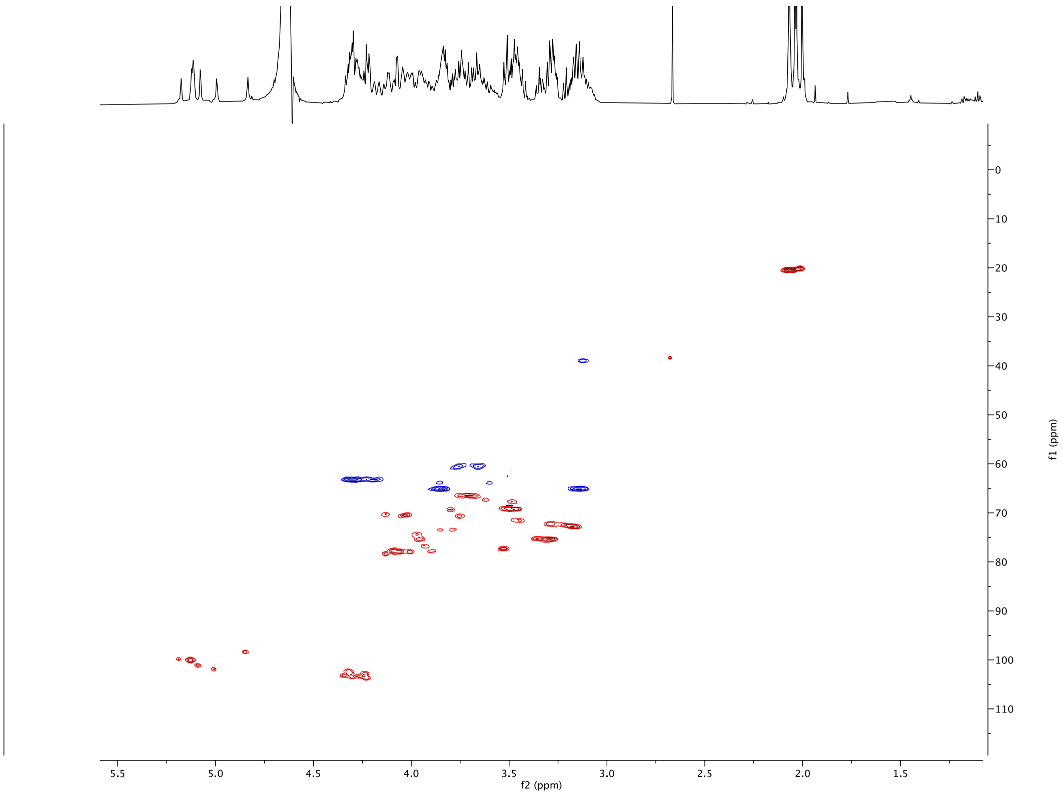

^{1}H-^{13}C HSQC NMR

Compound 18

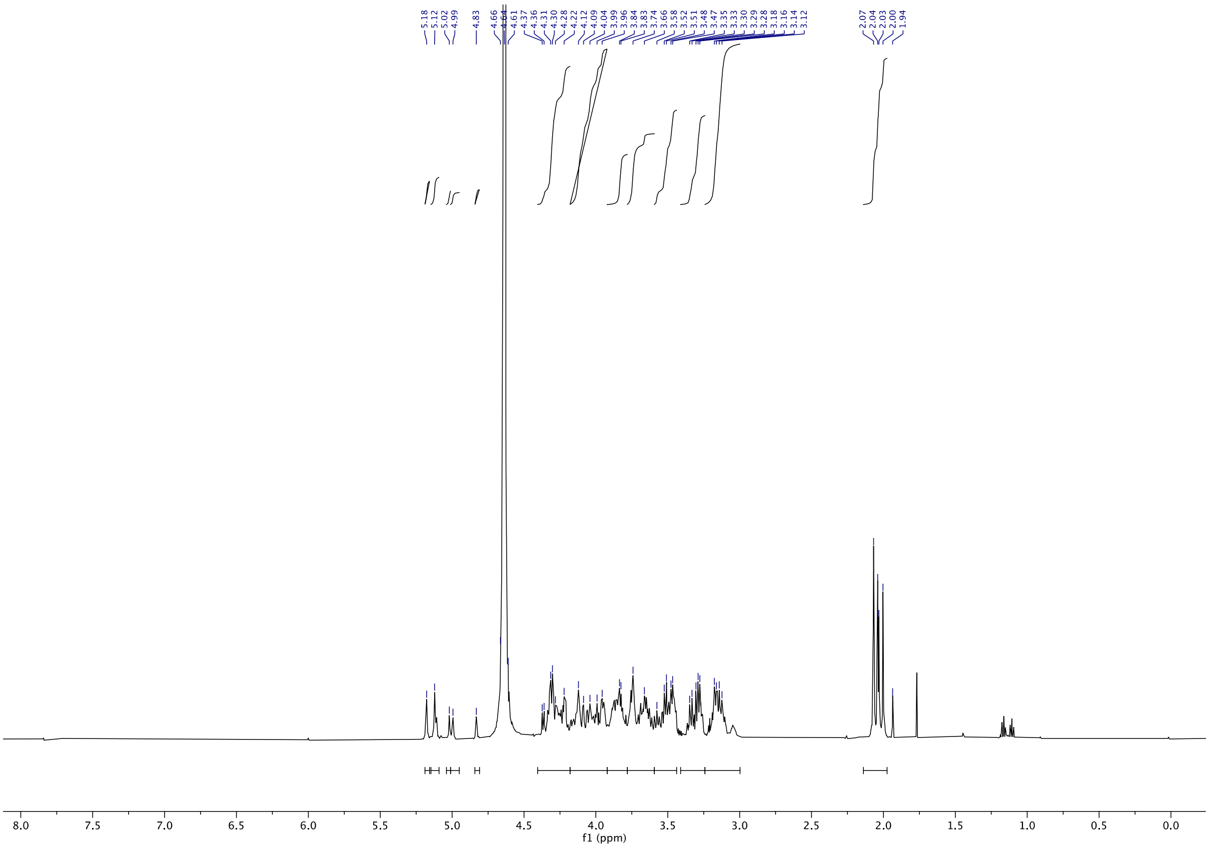

^{1}H NMR

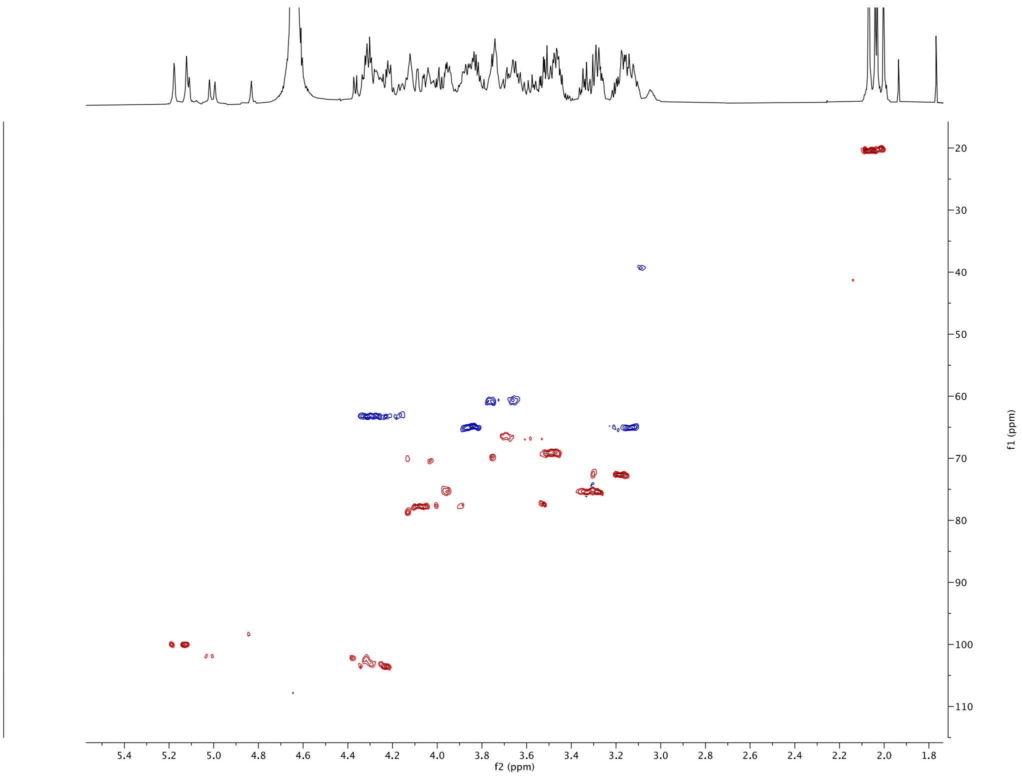

^{1}H-^{13}C HSQC NMR

Compound 26

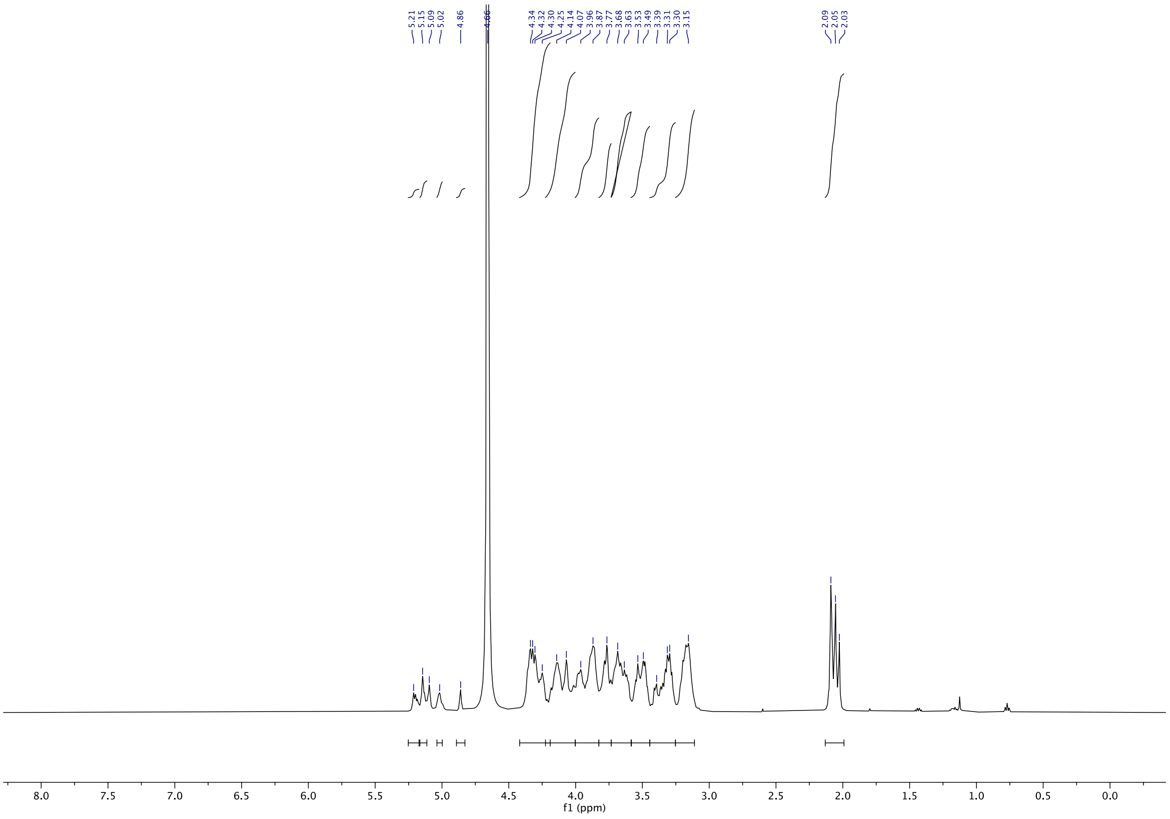

^{1}H NMR

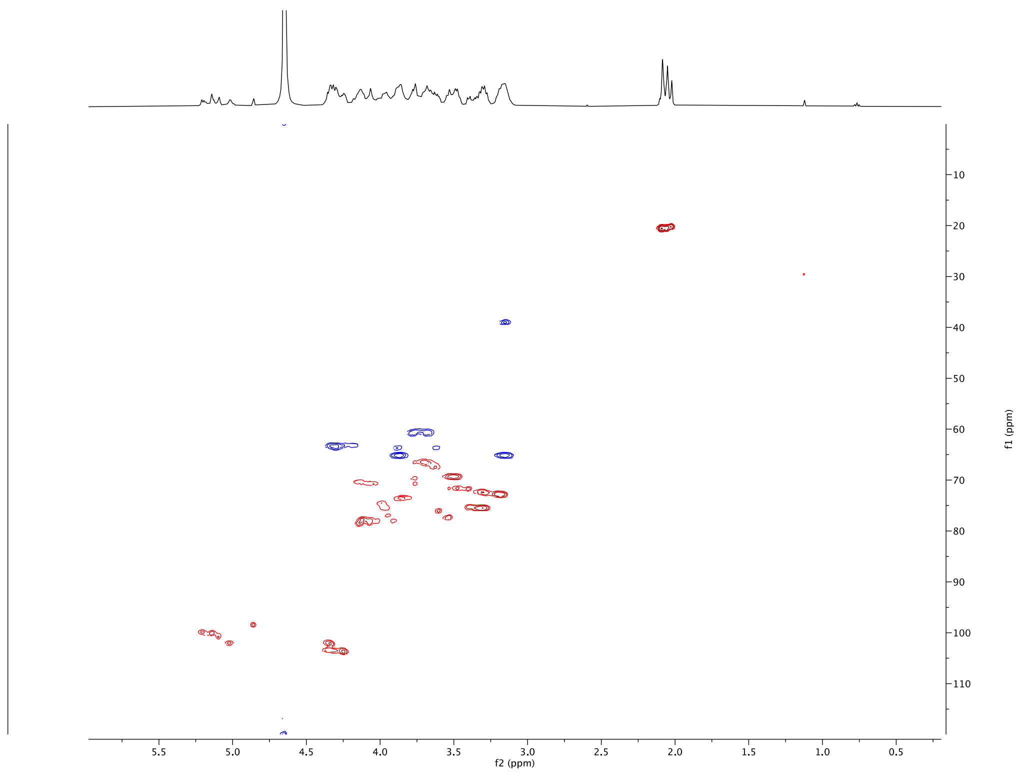

^{1}H-^{13}C HSQC NMR
